# A core outcome set for locoregional treatment reporting in neoadjuvant systemic breast cancer treatment trials

**DOI:** 10.1038/s41523-025-00824-w

**Published:** 2025-10-28

**Authors:** Shelley Potter, Kerry Avery, Rosina Ahmed, Jana de Boniface, Sanjoy Chatterjee, David Dodwell, Peter Dubsky, Jingjing Federmann, Sandra Finestone, Michael Gnant, Dominik Hlauschek, Hiroji Iwata, Michael Y. Jiang, Orit Kaidar-Person, Han-Byoel Lee, Mairead MacKenzie, Anne Meyn, Philip Poortmans, Fiorita Poulakaki, Andrea L. Richardson, Karla A. Sepulveda, Andrew J. Spillane, Alastair M. Thompson, Gustavo Werutsky, Philipp Wittmann, Jean L. Wright, Nicholas Zdenkowski, Katherine Cowan, Stuart A. McIntosh

**Affiliations:** 1 Bristol Surgical and Perioperative Care Complex Intervention Collaboration; Bristol Medical School, Bristol, UK; 2 Bristol Breast Care Centre, North Bristol NHS Trust, Bristol, UK; 3 Centre for Surgical Research, Bristol Medical School, Bristol, UK; 4 NIHR Bristol Biomedical Research Centre, University Hospitals Bristol and Weston NHS Foundation Trust and University of Bristol, Bristol, UK; 5 Tata Medical Centre, Kolkata, India; 6 Breast Unit, Capio St. Göran’s Hospital, Stockholm, Sweden; 7 Department of Medical Epidemiology and Biostatistics, Karolinska Institutet, Stockholm, Sweden; 8 University of Oxford, Oxford, UK; 9 Hirslanden Klinik St Anna, Lucerne, Switzerland; 10 University of Lucerne, Lucerne, Switzerland; 11 Austrian Breast & Colorectal Cancer Study Group, Vienna, Austria; 12 Patient advocate, California, United States of America; 13 Comprehensive Cancer Center, Medical University of Vienna, Vienna, Austria; 14 Aichi Cancer Centre, Nagoya, Japan; 15 Breast Radiation Unit, Sheba Medical Centre, Ramat Gan, Israel; School of Medicine, Faculty of Medical & Health Sciences, Tel-Aviv University, Tel-Aviv, Israel; GROW- Research Institute for Oncology and Reproduction, Maastricht University, Maastricht, Netherlands; 16 Dept. of Surgery, Seoul National University College of Medicine; Cancer Research Institute, Seoul National University; Biomedical Research Institute, Seoul National University Hospital, Seoul, Republic of Korea; 17 Independent Cancer Patients’ Voice, London, UK; 18 Department of Radiation Oncology, Iridium Netwerk, Wilrijk-Antwerp, Belgium; University of Antwerp, Faculty of Medicine and Health Sciences, Wilrijk-Antwerp, Belgium; 19 Europa Donna - The European Breast Cancer Coalition, Milan, Italy; 20 Breast Surgery Department, Athens Medical Centre, Maroussi, Greece; 21 John’s Hopkins Medicine, Baltimore, USA; 22 Baylor College of Medicine, Houston, TX USA; 23 University of Sydney, Sydney, Australia; 24 Latin American Cooperative Oncology Group, Porto Alegre, Brazil; 25 Department of Radiation Oncology at the University of North Carolina School of Medicine, Chapel Hill, NC USA; 26 University of Newcastle, Newcastle, Australia; 27 Katherine Cowan Consulting, Thame, UK; 28 Patrick G Johnson Centre for Cancer Research, Queen’s University Belfast, Belfast, UK; 29 NHS Highland, Inverness, UK; 30 Rambam Health Care Campus, Haifa, Israel; 31 Mansoura University, Mansoura, Egypt; 32 Oxford University Hospitals NHS Trust, Oxford, UK; 33 Lucerne Cantonal Hospital and University of Bern, Bern, Switzerland; 34 Sanjay Gandhi Postgraduate Institute of Medical Sciences, Lucknow, India; 35 Miró Hospital Universitario Sant Joan De Reus, Reus, Spain; 36 Hospital Del Mar, Barcelona, Spain; 37 Alves Hospital Universitaria Evangelico Mackenzie, Curitiba, Brazil; 38 National Medical Research Center of Oncology named after N.N.Petrov, St Petersburg, Russia; 39 Mid And South Essex NHS Foundation Trust, Essex, UK; 40 Instituto Alexander Fleming, Buenos Aires, Argentina; 41 University Hospital 12 De Octubre, Madrid, Spain; 42 Acibadem University, Istanbul, Turkey; 43 University Of Perugia and Perugia General Hospital, Perugia, Italy; 44 Shaukat Khanum Memorial Cancer Hospital and Research Centre, Lahore, Pakistan; 45 University of Manchester, Manchester, UK; 46 Christian Medical College, Vellore, India; 47 University of Tsukuba, Tsukuba, Japan; 48 University Hospital Schleswig-Holstein Campus, Lübeck, Germany; 49 CISSS De Chaudiere-Appalaches, Ste Marie, Canada; 50 Royal Marsden NHS Foundation Trust, London, UK; 51 Careggi University Hospital, Florence, Italy; 52 Università Politecnica Delle Marche and AOU Delle Marche, Ancona, Italy; 53 Sheba Tel Hashomer Medical Center, Raman Gat, Israel; 54 Vajira Hospital, Bangkok, Thailand; 55 Klinikum Ernst Von Bergmann, Potsdam, Germany; 56 Cambridge University Hospitals NHS Foundation Trust, Cambridge, UK; 57 Helios University Hospital Wuppertal - University Witten/Herdecke, Wuppertal, Germany; 58 Maastricht University Medical Centre, Maastricht, The Netherlands; 59 Iuliu Hatieganu University of Medicine and Pharmacy, Cluj-Napoca, Romania; 60 Pederzoli Hospital Peschiera Del Garda, Peschiera Del Garda, Italy; 61 Mater Dei Hospital, Msida, Malta; 62 Northern Centre for Cancer Care, Newcastle-upon-Tyne, UK; 63 University of New Mexico, Alberquerque, NM USA; 64 Ospedale Multimedica Castellanza, Castellanza, Italy; 65 Hospital Infanta Cristina, Madrid, Spain; 66 Adana Kozan State Hospital, Kozan, Turkey; 67 HSHS StVincent Hospital/Crown, Green Bay, WI USA; 68 Newcastle Upon Tyne NHS Hospitals, Newcastle, USA; 69 Uniting Health, Brisbane, Australia; 70 Hospital Universitario De Jaen, Jaen, Spain; 71 Sakarya University Training and Research Hospital, Sakarya, Turkey; 72 Peterko Clinical Hospital Centre Rijeka, Rijeka, Croatia; 73 United Hospitals of Derby and Burton, Derby, UK; 74 Hospital de Clinicas Dr Manuel Quintela, Montevideo, Uruguay; 75 NYU Langone Perlmutter Cancer Center, New York, USA; 76 Prince Of Wales Hospital and the Royal Hospital for Women, Sydney, Australia; 77 University of Hong Kong/Queen Mary Hospital, Hong Kong, Hong Kong; 78 Apollo Multispecialty Hospital, Kolkata, India; 79 Ampuero Hospital Universitario Severo Ochoa, Leganés, Spain; 80 Melbourne University Department of Surgery, Melbourne, Australia; 81 Medical Oncology Unit, H.UVirgen De Las Nieves, Granada, Spain; 82 Instituto De Investigacion Biosanitaria Ibs. Granada, Granada, Spain; 83 University Of Cyprus Medical School and Bank of Cyprus Oncology Centre, Nicosia, Cyprus; 84 Allegheny Health Network, Pittsburgh, PA USA; 85 University Of Southampton, Southampton, UK; 86 Ludwig-Maximilians-University Hospital, Munich, Germany; 87 ICS Maugeri, Pavia, Italy; 88 Hospital Lusíadas Lisboa Oncology Center, Lisbon, Portugal; 89 Belfast Health and Social Care Trust, Belfast, UK; 90 Ospedale Di Bolzano Azienda Sanitari Alto Adige, Bolzano, Italy; 91 Hospital Universitario Canarias, Santa Cruz de Tenerife, Tenerife Spain; 92 ASL Napoli 3 Sud, Naples, Italy; 93 European Medical Center, Moscow, Russia; 94 Swansea Bay University Health Board, Swansea, UK; 95 Université Libre De Bruxelles, Hôpital Universitaire De Bruxelles, Institut Jules Bordet, Brussels, Belgium; 96 Hospital Universitario Ramón Y Cajal, Madrid, Spain; 97 Instituto Catalan Oncologia, Girona, Spain; 98 University Of New South Wales School Of Clinical Medicine, Kensington, Australia; 99 Ghent University Hospital, Ghent, Belgium; 100 IRCCS San Raffaele Scientific Institute, Milan, Italy; 101 Quironsalud Madrid University Hospital, Madrid, Spain; 102 Medical University-Pleven, Pleven, Bulgaria; 103 Ankara Etlik City Hospital, Ankara, Turkey; 104 HFR Fribourg Hospital, Fribourg, Switzerland; 105 Metropolitan General Hospital of Athens, Athens, Greece; 106 St Luke’s Hospital, Thessaloniki, Greece; 107 Trinity St James’s Cancer Institute, Dublin, Ireland; 108 Cancer Trials Ireland, Dublin, Ireland; 109 Frauenklinik Ehingen, Ehingen, Germany; 110 University Witwatersrand, Johannesberg, South Africa; 111 Hospital Prof. Doutor Fernando Fonseca- ULS Amadora/Sintra, Amadora, Portugal; 112 Westmead Hospital, Westmead, Australia; 113 Breast-Center Zurich, Zurich, Switzerland; 114 Aberdeen Royal Infirmary, Aberdeen, UK; 115 Institut Català D’Oncología, Barcelona, Spain; 116 Ankara Training and Research Hospital, Ankara, Turkey; 117 Todua Clinic, Tblisi, Georgia; 118 Hospital Universitari Vall D’Hebron, Barcelona, Spain; 119 Instituto Do Cancer Do Estado De Sao Paulo - Faculdade De Medicina Da Universidade De Sao Paulo, Sao Paulo, Brazil; 120 Kettering General Hospital, Kettering, UK; 121 AOU Sassari, Sassari, Italy; 122 Alvaro Cunqueiro Hospital, Vigo, Spain; 123 Parc Tauli University Hospital, Sabadell, Spain; 124 Osakidetza, Bilbao, Spain; 125 AULSS 9 Scaligera, Verona, Italy; 126 São João University Hospital, Porto, Portugal; 127 Fondazione Policlinico Universitario Agostino Gemelli, Rome, Italy; 128 Instituto Nacional De Enfermedades Neoplasicas, Surquillo, Peru; 129 Specialty Surgical Oncology, Mumbai, India; 130 Università Della Svizzera Italiana, Lugano, Italy; 131 ASST Of Cremona/University of Trieste, Cremona, Italy; 132 University Hospital of Pisa, Pisa, Italy; 133 Oncosalud Auna, Lima, Peru; 134 Cleveland Clinic Abu Dhabi, Adu Dhabi, United Arab Emirates; 135 Gynäkologische Praxisklinik Hamburg Harburg, Hamburg, Germany; 136 Prof. Dr. Cemil Taşcıoğlu Hospital, Istanbul, Turkey; 137 Hadassah Hebrew University Medical Center, Jerusalem, Israel; 138 Centre Oscar Lambret, Lille, France; 139 Basavatarakam Indo American Cancer Hospital and Research Institute, Hyderabad, India; 140 Northumbria Healthcare NHS Foundation Trust, Newcastle Upon Tyne, UK; 141 Maidstone And Tunbridge Wells NHS Trust, Maidstone, UK; 142 University Hospital Basel, Basel, Switzerland; 143 McGill University, Montreal, Canada; 144 University Of Exeter, Exeter, UK; 145 All India Institute of Medical Sciences, Rishikesh, India; 146 Fondazione Poliambulanza, Brescia, Italy; 147 Shaikh Zayed Hospital Lahore and Rashid Latif Medical Complex, Lahore, Pakistan; 148 Hospital Universitario De Navarra, Pamplona, Spain; 149 Emory University School of Medicine, Atlanta, GA USA; 150 Health New Zealand, Canterbury, New Zealand; 151 Centre Hospitalier Universitaire de Liège, Liège, Belgium; 152 University Of Toronto, Toronto, Canada; 153 Wellington Hospital, Wellington, New Zealand; 154 Legacy Health Cancer Institute, Portland, Oregon, USA; 155 The Canberra Hospital, Canberra, Australia; 156 K M M Rajiv Gandhi Cancer Institute and Research Centre, Delhi, India; 157 Apollo Hospitals, Delhi, India; 158 Ondokuz Mayıs University General Surgery Department, Samsun, Turkey; 159 Pusan National University Hospital, Busan, South Korea; 160 Sri Shankara Cancer Hospital and Research Centre, Bengalaru, India; 161 Zonguldak Bulent Ecevit University, Zonguldak, Turkey; 162 Uppsala University, Uppsala, Sweden; 163 Khyber Medical College and Khyber Teaching Hospital, Peshawar, Pakistan; 164 Liga Nacional Contra El Cáncer and Instituto De Cancerología, Guatemala City, Guatemala; 165 Klinikum Rechts Der Isar, Tu Munich, Germany; 166 Peter Maccallum Cancer Centre, Melbourne, Australia; 167 Ushato Prof. Ivan Chernozemski, Sofia, Bulgaria; 168 Princess Alexandra Hospital, Harlow, UK; 169 National and Kapodistrian University of Athens, Athens, Greece; 170 Europa Donna Hellas, Athens, Greece; 171 Universitätsklinikum Hamburg-Eppendorf, Hamburg, Germany; 172 University Of Edinburgh, Edinburgh, UK; 173 Hampshire Hospitals, Winchester, UK; 174 Hospital Universitario De Badajoz, Badajoz, Spain; 175 Kyungpook National University Chilgok Hospital, Daegu, South Korea; 176 Gold Coast HHS, Southport, Australia; 177 Hospital Universitario Insular De Gran Canaria, Las Palmas de Gran Canaria, Spain; 178 Immanuel Klinik Märkische Schweiz, Buckow, Germany; 179 Catholic Hospital “Our Lady of Good Counsel”, Tirana, Albania; 180 University of Antwerp, Antwerp, Belgium; 181 Leeds Teaching Hospitals, Leeds, UK; 182 University of Glasgow, Glasgow, UK; 183 Insituto Portuguēs De Oncologia De Coimbra Francisco Gentil, E.P.E., Coimbra, Portugal; 184 University Medical Centre Groningen, Groningen, Netherlands; 185 Centro De Educación Médica E Investigaciones Clínicas, Buenas Aires, Argentina; 186 Gartnavel General Hospital, Glasgow, UK; 187 Center for Treatment and Cancer Research, Bogota, Colombia; 188 Rigshospitalet, Copenhagen, Denmark; 189 Bank Of Cyprus Oncology Centre, Strovolos, Cyprus; 190 Washington University School of Medicine, Washington DC, USA; 191 Hospital Sírio-Libanês, Sao Paulo, Brazil; 192 Mcgill University Jewish General Hospital, Montreal, Canada; 193 Hospital Universitario La Paz, Madrid, Spain; 194 Lausanne University Hospital, Lausanne, Switzerland; 195 Broomfield Hospital, Mid and South Essex NHS Trust, Chelmsford, Essex, England UK; 196 Kyoto University, Kyoto, Japan; 197 Helios Klinikum Berlin Buch Breast Center, Berlin, Germany; 198 University of Florence, AOU Careggi, Florence, Italy; 199 University Hospital of Modena, Modena, Italy; 200 Helsinki University Hospital, Helsinki, Finland; 201 McGill University Health Center, Montreal, Canada; 202 Azienda Sanitaria Universitaria Friuli Centrale, Udine, Italy; 203 HM Hospitales, Madrid, Spain; 204 University of Sheffield, Sheffield, UK; 205 European Institute of Oncology, IRCCS, Milan, Italy; 206 University Hospital Zielona Gora Poland Clinic of General Surgery and Surgical Oncology, Zielona Gora, Poland; 207 Hawler Medical University, Erbil, Iraq; 208 Hospital Universitario Lucus Augusti, Lugo, Spain; 209 Yokohama City University Medical Center, Yokohama, Japan; 210 Westchester Medical Center, Valhalla, New York, USA; 211 UMC Bezanijska Kosa, Belgrade, Serbia; 212 Athens Medical Group, Athens, Greece; 213 North West Cancer Centre, Altnagelvin Hospital, Londonderry, UK; 214 Södersjukhuset, Stockholm, Sweden; 215 Sahlgrenska University Hospital, Gothenburg, Sweden; 216 Hospital Virgen De Los Lirios, Alcoy, Spain; 217 IASO Maternity Hospital, Marusi, Greece; 218 Regional And Virgen De La Victoria University Hospitals, Malaga, Spain; 219 IRCCS Azienda Ospedaliero Universitaria Di Bologna, Bologna, Italy; 220 Xarxa Santa Tecla Sanitària, Tarragona, Spain; 221 Hospital Clínico Universitario Virgen De La Arrixaca, Murcia, Spain; 222 Maria Sklodowska-Curie National Research Institute of Oncology, Warsaw, Poland; 223 Tumor Zentrum Aarau, Aarau, Switzerland; 224 Huntsman Cancer Institute, Salt Lake City, UT USA; 225 Breast Unit, Sant’Anna Hospital, Turin, Italy; 226 European Organisation for Research and Treatment of Cancer Headquarters, Brussels, Belgium; 227 Real Hospital Beneficência Portuguesa Pernambuco, Recife, Brazil; 228 Minsk City Clinical Oncology Centre, Minsk, Belarus; 229 Kantonsspital St. Gallen, St. Gallen, Switzerland; 230 Cancer Institute (W.I.A), Adyar, Chennai, India; 231 Square Hospitals Ltd, Dhaka, Bangladesh; 232 K S Hegde Medical Academy, Nitte University, Mangalore, India; 233 University of Ljubljana, Ljubljana, Slovenia; 234 Hygeia Hospital, Athens, Greece; 235 ACT Health, Australian National University, Canberra, Australia; 236 Fiona Stanley Hospital, Murdoch, Australia; 237 Santa Casa De Porto Alegre, Porto Alegre, Brazil; 238 Grupo Oncologico Cooperativo Chileno De Investigacion, Santiago, Chile; 239 Oslo University Hospital, Oslo, Norway; 240 University of Naples Federico II, Naples, Italy; 241 Centro Integral Oncológico Clara Campal, Madrid, Spain; 242 East Cheshire NHS Trust, Macclesfield, UK; 243 Centre Du Sein, Fribourg, Switzerland; 244 ASST Sette Laghi Di Varese, Varese, Italy; 245 Clinica Universidad De Navarra, Madrid, Spain; 246 The Netherlands Cancer Institute, Amsterdam, Netherlands; 247 AZ Turnhout, Turnhout, Belgium; 248 Samsung Medical Center, Seoul, South Korea; 249 Karolinska Institutet Stockholm, Stockholm, Sweden; 250 Colchester Hospital, Colchester, UK; 251 Centro Medico Privado “Cemaic”/Hospital Oncologico De Cordoba, Cordoba, Argentina; 252 Siriraj Hospital - Mahidol University, Bangkok, Thailand; 253 Mysore Medical College and Research Institute, Mysore, India; 254 Ain Shams University, Cairo, Egypt; 255 Kasturba Medical College and Hospital, Manipal, India; 256 Dr G. Stransk University Hospital, Pleven, Bulgaria; 257 University Hospitals of Derby and Burton, Derby, UK; 258 West Lisbon Local Health Unit, Lisbon, Portugal; 259 Hospital Santa Catarina Paulista, São Paulo, Brazil; 260 University Hospitals Sussex NHS Foundation Trust, Brighton, UK; 261 Vedanat Medical Research Foundation, Balco Medical Centre, Naya Raipur, India; 262 Mater Hospital, Sydney, Australia; 263 University of Colorado Anschutz Medical Center, Aurora, CO USA; 264 University of North Carolina, Chapel Hill, NC USA; 265 Lawatsch Med Universität Graz Strahlentherapie, Graz, Austria; 266 Saint Paul’s Hospital Millennium Medical College, Addis Ababa, Ethiopia; 267 Health New Zealand, Wellington, New Zealand; 268 Vardhman Mahavir Medical College and Safdarjung Hospital, New Delhi, India; 269 U.O.C.Senologia Chirurgica ASL2 Abruzzo, Abruzzo, Italy; 270 CHU UCL Namur Sainte Elisabeth, Namur, Belgium; 271 Hospital Do Câncer De Londrina, Londrina, Brazil; 272 Acibadem Altunizade Hospital, Istanbul, Turkey; 273 Peninsula Health, Frankston, Australia; 274 Queen Mary University of London, London, UK; 275 Athens Medical Centre, Athens, Greece; 276 Geneva University Hospital, Geneva, Switzerland; 277 Gentofte Hospital, Hellerup, Denmark; 278 Cancer Institute Hospital of JFCR, Tokyo, Japan; 279 University College London, London, UK; 280 Örebro University, Örebro, Sweden; 281 Zuyderland MC, Heerlen, Netherlands; 282 Leiden University Medical Center, Leiden, Netherlands; 283 Technical Medical Centre, University of Twente, Enschede, The Netherlands; 284 Netherlands Comprehensive Cancer Organisation, Utrecht, The Netherlands; 285 Cliniques Universitaires Saint-Luc, Woluwe-Saint-Lambert, Belgium; 286 Hospital Meixoeiro, Vigo, Spain; 287 Kasturba Medical Colle, Manipal Academy of Higher Education, Manipal, India; 288 Ιpswich Ηospital, Ipswich, UK; 289 Hospital Clinic de Barcelona, Barcelona, Spain; 290 Aga Khan University Hospital, Karachi, Pakistan; 291 Sault Area Hospital, Sault Ste Marie, Canada; 292 Tata Memorial Centre, Mumbai, India; 293 Alfred Health, Melbourne, Australia; 294 Stanford University, Stanford, CA USA; 295 Gothenburg University, Gothenburg, Sweden; 296 Cross Cancer Institute, Edmonton, Canada; 297 Yadav NSCB Medical College, Jabalpur, India; 298 University of Cyprus, Nicosia, Cyprus; 299 Jeonbuk National University Medical School, Jeonju, South Korea; 300 Silesian University, Opava, Czech Republic; 301 Hospital General De Granollers, Granollers, Spain; 302 University Hospital “Mother Teresa”, Tirana, Albania; 303 Radojevic University of Kragujevac, Kragujevac, Serbia; 304 Marengo Cims Hospital, Ahmedabad, India

**Keywords:** Breast cancer, Outcomes research, Clinical trial design

## Abstract

Accurate information about locoregional breast cancer treatments following neoadjuvant systemic therapy (NST) is essential for meaningful interpretation of oncological outcomes but reporting is currently poor. We developed a core outcome set (COS) to improve the quality and consistency of locoregional outcome reporting in breast cancer NST trials. The COS was developed in three phases according to COS-STAD guidance, with the generation of a list of relevant outcome domains, prioritisation of outcomes through two rounds of an international online multi-stakeholder Delphi survey and a consensus meeting. 159 unique locoregional outcomes were classified into 101 outcome domains for inclusion in the Delphi survey, which was completed by 470 international professionals. The final 15-item COS, which included the pre-NST surgical plan, details of surgery performed following completion of treatment and details of radiation therapy, was agreed at an in-person consensus meeting. Widespread COS implementation will improve the quality and value of future NST trials.

## Introduction

Neoadjuvant (or primary) systemic therapy (NST) is increasingly used in the treatment of early breast cancer as it confers multiple potential benefits for patients^1^. These include permitting downstaging of locoregional treatments such as surgery^2,3^ and radiation therapy (RT)^4^ in those who have a good treatment response and allowing response-adapted tailoring of adjuvant systemic therapies^5,6^ with escalation or de-escalation of treatments to optimise both oncological outcomes and quality of life. As treatment response is also highly prognostic on an individual level^7,8^, NST has become the standard of care for specific disease subtypes^9–11^.

Locoregional therapies, however, remain an integral component of the effective management of early breast cancer and accurate information about the surgery and RT performed following completion of NST is essential for the meaningful interpretation of trial results. The quality and consistency of locoregional treatment reporting in NST trials, however, is poor and needs to be improved^12,13^. This was first highlighted in 2018 by the Early Breast Cancer Trialists Collaborative Group (EBCTCG) meta-analysis of the outcomes of neoadjuvant and adjuvant chemotherapy, which included ten trials involving 5,250 women and suggested a higher rate of locoregional recurrence (LRR) in patients receiving NST^12^. This difference in LRR did not translate into significant differences in either distant recurrence or overall survival between the groups and the authors commented that the poor quality of locoregional treatment reporting in the included trials precluded meaningful interpretation of the results^12^. While it could be argued that the trials included in the EBCTCG meta-analysis were largely historic and reporting will have improved over time, a recent systematic review of contemporary NST studies suggests that locoregional treatment reporting remains heterogenous and inconsistent^13^. This review which included 137 NST trials involving 575,531 women published between 2018 and 2023 showed that most studies continued to report minimal, if any information regarding the surgery and RT performed^13^. Not only does this hamper accurate interpretation of key oncological outcomes such as LRR, it may also explain the consistent failure to translate breast cancer downstaging into a reduction in the extent of surgery in both trials and real-world cohorts, where significant proportions of patients continue to undergo mastectomy despite achieving a complete pathological response (pCR)^2,3,14^.

One solution to addressing poor and inconsistent outcome reporting in NST trials may be to develop and implement a core outcome set (COS), ‘*an agreed standardized set of outcomes that should be measured and reported, as a minimum, in all clinical trials’*^15^. For this approach to be successful, however, it is vital that the international breast cancer research community is engaged in the development of a COS and committed to future implementation. The aim of the PRECEDENT (imProving REporting of loCoregional therapies in nEoaDjuvant brEast caNcer Trials) project was therefore to work with key international stakeholders to develop a COS for locoregional treatment measurement and reporting in NST trials.

## Results

### Phase 1: Generation of a list of outcome domains

A total of 747 locoregional outcomes (510 surgical and 237 RT related) identified from the systematic review^13^ were categorised into 68 surgical and 28 RT outcome domains. Focused discussion with key stakeholders identified one additional surgical and four additional RT outcome domains for inclusion. Following discussion and iterative refinement by the steering group, a total of 101 outcome domains (69 surgical and 32 RT related) were carried forward for survey prioritisation.

### Phase 2: Outcome domain prioritisation

The 101 outcome domains grouped thematically into nine surgery and four RT sections and formatted into 101 questionnaire items for inclusion in the international Delphi survey.

A total of 470 healthcare professionals prioritised at least one COS item in Round 1 and there was good multidisciplinary representation from the surgical (*n* = 206, 43.8%), medical (*n* = 144, 30.6%) and radiation (*n* = 98, 20.8%) oncology communities (Table 1). Respondents were predominantly from Europe (*n* = 274, 58.3%) but there was broad geographical representation reflecting good international engagement with the survey. Most participants were research active (*n* = 437, 93.0%), experienced clinicians (>10 years at consultant/attending level *n* = 307, 65.3%) working in Academic/University/Teaching hospital settings (*n* = 338, 71.9%) and over half (*n* = 248, 55.8%) identified as female.

**Table 1:** Demographics of Delphi participants

	Round 1 (*N* = 470)	Round 2 (*N* = 336)
**Role**^**a**^		
Surgeon^a^ Medical Oncologist Radiation Oncologist^c^ Other^d^	206 (43.8) 144 (30.6) 98 (20.8) 22 (4.7)	153 (45.5) 90 (26.8) 77 (22.9) 16 (4.8)
**Age**		
<30 30-39 40-49 50-59 60 or over Prefer not to say/not reported	5 (1.1) 96 (20.4) 158 (33.6) 146 (31.1) 56 (11.9) 9 (1.9)	4 (1.2) 69 (20.5) 118 (35.1) 99 (29.5) 39 (11.6) 7 (2.1)
**Geographical location of practice**		
Europe Asia North America South America Australia/New Zealand Middle East Africa	274 (58.3) 60 (12.8) 46 (9.8) 38 (8.1) 32 (6.8) 14 (3.0) 6 (1.3)	209 (62.2) 45 (13.4) 30 (8.9) 19 (5.7) 20 (6.0) 9 (2.7) 4 (1.2)
**Gender**		
Female Male Prefer not to say/not reported	248 (55.8) 213 (45.2) 9 (1.9)	173 (51.5) 155 (46.1) 8 (2.4)
**Years of experience at Consultant/Attending level**		
< 5 years 5–10 years >10 years Prefer not to say/ not reported	64 (13.6) 94 (20.0) 307 (65.3) 5 (1.1)	47 (14.0) 69 (20.5) 216 (64.3) 4 (1.2)
**Main type of practice**		
Academic/University/Teaching Hospital Public Hospital Private Hospital Other	338 (71.9) 58 (12.3) 56 (11.9) 18 (3.8)	246 (73.2) 38 (11.3) 40 (11.9) 12 (3.6)
**Research active**^e^	437 (93.0)	316 (94.1)

^a^If more than one role stated, respondents were classified according to involvement in locoregional breast cancer treatments.

^b^13 surgeons also prescribed chemotherapy.

^c^Includes 1 Consultant Therapeutic Radiographer (RTT) and 18 respondents who prescribed chemotherapy and gave radiation therapy.

^d^Radiologist *n* = 4; pathologist *n* = 2; non-clinical role *n* = 6, not stated = 3.

^e^Defined broadly as designing, leading or recruiting to clinical trials or other research studies.

A total of 336 (71.5%) Round 1 participants (153/206 (74.3%) surgeons, 90/144 (62.5%) medical oncologists and 77/98 (78.6%) radiation oncologists) prioritised at least one COS item in Round 2. Respondent demographics were similar between rounds (Table 1).

Scores for each outcome domain following Round 2 are summarised by stakeholder group in Supplementary Table 1. Applying the revised definitions of consensus (Table 2), 31 outcome domains were scored ‘consensus in’ (scored ‘very important’ by ≥70% surgeons and/or radiation oncologists and/or other professionals). These were reviewed and, where thematically relevant, combined into 15 summary outcome domains that were carried forward for review and ratification at the consensus meeting (Supplementary Table 2). Ten outcome domains were scored as ‘no consensus’ (scored very important by 60–70% of surgeons and/or radiation oncologists) so were carried forward for discussion and voting at the meeting. The remaining 60 outcome domains were scored ‘consensus out’ (scored ‘very important’ (7–9) by <60% surgeons and/or radiation oncologists) and were carried forward to the consensus meeting to ratify their exclusion (Supplementary Table 1).

**Table 2:** Definitions of consensus

Category	Original (Rounds 1 and 2)		Revised (Round 2 only)		Consensus Meeting	
	Definition	Action	Definition	Action	Definition	Outcome
‘Consensus in’	Scored as very important (7–9) by ≥70% *and* not important (1–3) by <15% of any stakeholder group (surgeons and/or radiation oncologists and/or others)	Domain retained for next survey round/consensus meeting	Scored as very important (7–9) by ≥70% of any stakeholder group (surgeons and/or radiation oncologists and/or others)	Domain reviewed and thematically similar outcomes merged into broader summary outcomes for review/ratification at consensus meeting	Scored as ‘very important’ by ≥70% meeting participants^a^	Summary domain included in final COS following ratification*
‘Consensus out’	Scored as not important (1–3) by ≥70% *and* very important (7–9) by <15% any stakeholder group (surgeons and/or radiation oncologists and/or others)	Domain discarded after Round 2 (to be ratified at consensus meeting)	Scored as very important by <60% of locoregional therapists (surgeons and/or radiation oncologists)	Same as original	Scored as ‘very important’ by <70% of meeting participants	Domain not included in final COS, following ratification
‘No consensus’	None of the criteria above are met	Domain retained for next survey round/consensus meeting	Neither of the above criteria are met	Same as original	Same as Round 2	Included in final COS if voted ‘very important’ by ≥70% meeting participants** Not included in final COS if voted ‘very important’ by <70% meeting participants, following ratification

^*^either as originally worded for revised/merged with another domain(s), **may be included as separate outcome domain or merged with another summary domain.

^a^For ratification agreement was required from ≥70% meeting participants.

#### Phase 3: Consensus meeting

Twenty-three experienced professionals including seven surgeons, 13 radiation oncologists, two medical oncologists, a radiologist and five patient advocates with broad international representation (Europe *n* = 18, North America *n* = 4; Middle East 2, Asia *n* = 3; Australia *n* = 1) attended the in-person consensus meeting. Following discussion, participants agreed to include the 15 ‘consensus in’ summary outcome domains in the final COS but proposed that the ‘side effects of RT’ domain was amended to reflect the morbidity associated with locoregional treatments (surgery and RT) more broadly. Minor revisions of outcome domain wording were discussed and ratified. Following discussions and voting, none of the 10 ‘no consensus’ items met the threshold for inclusion (≥70% meeting participants scoring outcome ‘very important’) in the COS so these and the 60 ‘consensus out’ items were excluded. The final revised 15-item COS for locoregional outcome reporting in NST studies was agreed and ratified (Table 3).

**Table 3:** Final core outcome set for locoregional treatment outcome reporting in breast cancer neoadjuvant systemic therapy trials

No	Core outcome set domain
1	The type of breast and axillary surgery planned before starting NST
*2*	*The proportion of patients who did not have surgery after NST due to disease progression, treatment toxicities or other comorbidities*
3	The number/proportion of patients *with a complete response to NST* not having surgery to the breast and/or axilla, and how response was assessed
4	How response to NST in the breast and axilla was assessed
5	Type of *initial* breast and axillary surgery performed after NST
6	*Proportion of patients with involved margins after initial and final surgery, and number of procedures required (and recorded definition of clear margins at a****trial level***^***a***^*)*
7	Total number of excised and involved axillary lymph nodes, with extent of involvement
8	Further axillary treatment in patients with ypN+ disease after sentinel node biopsy/targeted axillary dissection (SLNB/TAD)
9	Proportion of patients in whom breast and/or axillary surgery was downstaged
10	The proportion of patients having radiation therapy
11	Indications for radiation therapy ***at trial level***^***a***^ *(recorded in protocol and reported)*
12	Details of breast/chest wall and nodal targets
13	Details of dose and fractionation to breast/chest wall and nodal areas
14	Receipt of boost; indications for boost (***at trial level***^***a***^)
*15*	*Morbidity of locoregional treatments (short and long term as defined in protocol)*

*Denotes revised wording agreed and ratified at consensus meeting;* *NST* neoadjuvant systemic therapy; ^a^should be described/recorded a priori in the trial protocol.

## Discussion

This robust international consensus process has developed a core outcome set for locoregional outcome reporting in breast cancer neoadjuvant systemic therapy trials. It includes 15 outcomes that all key stakeholders think are essential to measure and report as a minimum in all future NST studies. Surgical outcomes include the type of breast and axillary surgery performed; how response to NST is assessed; the adequacy of surgery performed, the proportion of patients who had their breast or axillary surgery downstaged and how many avoided surgery due to an exceptional treatment response. Radiation therapy outcomes included receipt of RT and details of target volumes, dose and fractionation. Short and long-term locoregional treatment morbidity (as defined in the protocol) was also a key outcome. Reporting this essential information regarding locoregional therapy in NST trials will improve their quality and value, ensure that results can be translated into practice and important oncological outcomes such as locoregional recurrence can be interpreted with confidence.

Previous attempts have been made to standardise outcome reporting^16,17^, but these have not been widely implemented into practice. One reason for this may be that these recommendations were developed based on expert opinion without engagement of the wider breast cancer community. The PRECEDENT COS has specifically been developed in collaboration with key professional stakeholders and experienced patient advocates involved in breast cancer trials worldwide using established methods for consensus to promote engagement and future implementation of the COS from the start^18^. The COS developed using this collaborative, inclusive and methodologically robust approach, therefore includes outcomes that are meaningful to the international breast cancer community, who should then feel empowered to promote the uptake and implementation of the COS in future NST studies.

Complexity may also represent a barrier to implementation. Several international groups have robustly developed detailed recommendations for locoregional therapy reporting^19^, but collecting significant amounts of additional data is time consuming and expensive. This is a specific concern in the NST setting where many trials are designed and funded by the pharmaceutical industry to meet regulatory requirements, often without the involvement of surgeons or radiation oncologists to emphasise the importance and relevance of reporting locoregional treatments. A COS is, by definition, an essential set of outcomes that are important to all key stakeholders that should be reported *as a minimum* in all future NST trials. This minimalistic approach to locoregional outcome selection may represent a more acceptable strategy to improving outcome reporting in NST trials but engagement with funders, trialists and regulators including the US Food and Drug Administration and the UK Medicines and Healthcare products Regulatory Agency will be essential to ensure robust locoregional outcome reporting in all future studies of drugs used in the neoadjuvant setting.

It is also important to consider how the purpose of this COS differs from other breast cancer metrics, such as quality indicators (QIs)^20^. Core outcome sets aim to improve the quality and consistency of outcome reporting in trials, facilitate data synthesis and reduce research waste^21^. They represent a *minimum* reporting standard in clinical trials; they do not restrict the number of outcomes that can be measured or imply that other outcomes are not important. Quality indicators (QIs), by contrast, are used to monitor the quality of clinical care in routine practice. Unlike core outcome sets, which are limited to a small number of essential outcomes that must be reported^18^, there is no restriction on the number of QIs that may be proposed, as these should, by definition, cover all aspects of the treatment pathway. This difference in function explains why the COS and published QIs differ. Outcomes such as ‘time from completion of NST to surgery’ for example are not considered essential to include in the COS for locoregional outcome reporting in NST trials, but are a key quality indicator^20^ as delays in surgery adversely impact oncological outcomes^22^. By contrast, the proportion of patients with an exceptional response post NST who are able to omit or avoid surgery is a core outcome that should be reported in all NST trials but is not currently considered a quality indicator used to measure the quality of the care received.

This work has limitations that should be considered. Firstly, given the complexity of the topic, the consensus process was undertaken in English. This may have limited the participation to individuals with high levels of English proficiency. However, the key stakeholders for this COS were experienced breast cancer trialists. English is the primary language for scientific communication so restricting the process to English would be unlikely to significantly impact the results.

A robust international engagement strategy was embedded in the project a priori to optimise future implementation. Central to this strategy was a partnership with the BIG-NCTN network and the recruitment of multidisciplinary representatives of the major international breast cancer research groups to the steering group to provide access to global speciality networks. This strategy was partially successful, with excellent engagement with professionals from Europe and good international participation overall. There were, however, notably fewer Delphi participants from North America (<10%) with less than two thirds of these individuals participating in both survey rounds. Reasons for this are unclear but there was excellent international representation at the consensus meeting to ensure the final COS was acceptable in different healthcare settings. Similarly, despite strong steering group representation from the radiation oncology community, proportionally fewer radiation oncologists participated in the Delphi. This group, however, were the most likely to complete both survey rounds and were well represented at the consensus meeting, providing confidence that the RT outcomes included in the COS were the most important to radiation oncologists more broadly.

Finally, although patients did not participate in the Delphi, every effort was made to ensure that both the project and the final COS were patient-focused. This was achieved from the start, with experienced patient advocate members of the steering group actively participating in discussions about the project and helping to shape the Delphi survey. Strong international patient representation at the consensus meeting ensured the patient perspective was central to discussions. The final COS, therefore, included outcomes that were equally important to patients as well as professionals.

For the newly developed COS to improve the quality and consistency of locoregional outcome reporting in breast cancer NST trials, however, it will need to be accepted and effectively implemented into practice. To facilitate this process, the PRECEDENT group have worked with experienced trialists and data managers from the Austrian Breast and Colorectal Cancer Study Group to operationalise the 15-item COS into an easy-to-use, practical case report form (CRF) that can be embedded in all future NST trials (Supplementary Materials 3). The CRF is available in several electronic formats that can be uploaded into trial databases, minimising the effort required to include the COS in a trial dataset. The CRF includes clear, well-defined outcomes that will allow all trialists to consistently collect the same, minimum locoregional treatment dataset across all future NST studies; promote standardised implementation of the COS in future trials and facilitate data pooling and future meta-analysis. It may also enable streamlined data collection using artificial intelligence-driven real-world datasets as research methods continue to evolve^23,24^. Work is now needed to engage funders, study sponsors and regulatory agencies worldwide in the need to mandate inclusion of the locoregional reporting COS in all future NST studies, improving their quality and value to patients, trialists and the international breast cancer community as part of our ongoing efforts to improve care.

## Methods

The COS was developed using international consensus methods in accordance with Core Outcome Measures in Effectiveness Trials (COMET)^18^ and Core Outcome Set-STAndards for Development (COS-STAD) guidelines^25^. The full protocol including key definitions of ‘locoregional therapy’ and ‘NST’ has been published previously^26^ and the study prospectively registered on the COMET website (https://www.comet-initiative.org/Studies/Details/2854).

**Fig. 1:**
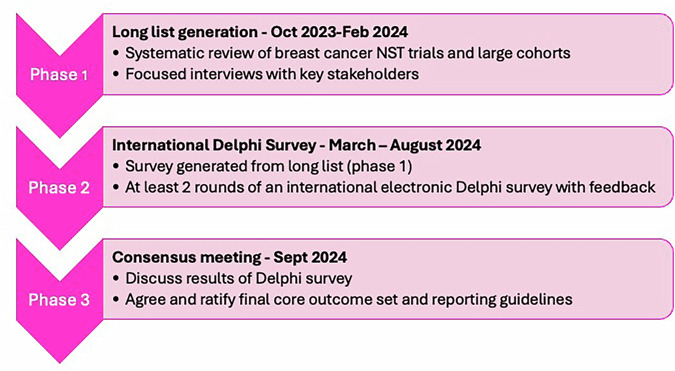
PRECEDENT COS development process.

The PRECEDENT project comprised three phases: 1) Generation of a list of outcomes from the literature and interviews with key stakeholders; 2) Outcome prioritisation in an international Delphi survey; 3) A consensus meeting to agree the final COS (Fig. 1).

### Stakeholder, patient and public engagement

This project was developed and delivered in conjunction with the Breast International Group (BIG) and the North American National Cancer Institute National Clinical Trials Network (NCI-NCTN) (BIG-NCTN collaborative group). A collaborative approach was chosen to promote engagement with the international breast cancer research community, to ensure the future COS would be broadly applicable across all healthcare settings and to optimise future adoption and implementation. An expert steering group, including representatives from the major breast cancer research networks across the world and patient advocates, was convened to provide overall oversight of the project. The steering group comprised 23 key stakeholders with expertise in breast cancer NST trials including patients, surgeons, medical and radiation oncologists, radiologists, pathologists, trialists and methodologists. Patient advocates advised on all aspects of the project and attended the consensus meeting to ensure the final COS included outcomes that were relevant and meaningful to patients.

### Phase I: Generation of a list of outcomes

A systematic review of primary NST studies published between 2018 and 2023 was used to identify locoregional outcome domains for inclusion in the Delphi survey^13^. Outcomes were defined broadly and included traditional outcomes such as complications following surgery, as well as additional factors which would influence the interpretation of locoregional recurrence, such as the adequacy of surgical resection or target volumes irradiated. All outcomes were extracted verbatim by two reviewers, with discrepancies resolved by discussion. The long list was then categorised into outcome domains by the study team (MJ/SP/SAMcI) and discussed with a purposively selected group of expert stakeholders (surgeons and radiation oncologists) to review the comprehensiveness of the list and identify additionally relevant domains in a series of brief focused interviews. The final long list of outcome domains was reviewed and refined by the steering group prior to outcome prioritisation.

### Phase II: Outcome domain prioritisation

Sequential rounds of an international multistakeholder Delphi survey were used to prioritise the outcome domains for inclusion in the COS.

Outcome domains were operationalized into survey questionnaire items for inclusion in the Delphi survey. Questions were written in English and reviewed by the international steering group to ensure the terminology used was appropriate and comprehensible to participants from different geographical regions. The survey was piloted with a small group of surgeons and radiation oncologists who were not involved in the project to ensure face validity prior to launch.

Respondents were asked how important it would be to include each outcome domain in a COS on a nine point Likert scale ranging from 1 (not important) to 9 (extremely important) based on the Grading of Recommendations Assessment, Development and Evaluation (GRADE) scale for including items in the final COS^27^.

Key stakeholders were professionals involved in breast cancer NST trials worldwide. This included surgeons, radiation and medical oncologists, radiologists and methodologists. Steering group members circulated the Round 1 survey widely through their professional networks, breast cancer groups and social media to promote participation and international engagement.

Following extensive discussion with the steering group, including patient advocates, it was agreed that patients would not be recruited to participate in the Delphi survey. This was due to the highly technical nature of the surgery and radiation oncology outcome domains. An alternative patient involvement strategy was devised. This included active patient advocate involvement in survey development including review of the long list to ensure its comprehensiveness and patient participation in the consensus meeting to ensure the final COS maintained a patient focus. To enable their full participation in the consensus meeting, preparatory briefing meetings were held with attending patient advocates to answer questions and explain the processes/methods that would be involved.

Participants completed two sequential survey rounds. Round 1 was open between 03/28/24 and 06/13/2024 and Round 2 between 06/26/24 and 08/02/2024. The survey was administered online using REDCap^28^ data capture software to facilitate international engagement and collaboration. Respondents were categorised into three key stakeholder groups: (i) surgeons, (ii) radiation oncologists and (iii) other professionals as the views of locoregional therapists were considered particularly relevant to consensus development. All Round 1 participants were invited to participate in Round 2. In Round 2, each survey item was accompanied by anonymised feedback about how that item was scored in Round 1, with the aim of promoting consensus across groups whose views may differ^18,29,30^. The feedback included (i) the participant’s own Round 1 score, (ii) a table summarising the median scores for each key stakeholder group and (iii) a histogram showing the distribution of scores by the stakeholder group. All items were retained between survey rounds, allowing participants to review their score and re-score each item, considering the feedback received. It was agreed a priori that a third survey round may be required if sufficient consensus was not achieved following Round 2.

Simple summary statistics were used to describe the participants’ characteristics in each survey round.

Following Round 2, the proportion of participants in each key stakeholder group scoring each item as ‘not important’ (score 1–3); ‘equivocal’ (score 4–6) and ‘very important’ (score 7–9) was calculated and each item categorised as ‘consensus in’, ‘consensus out’ or ‘no consensus’.

Consensus was defined a priori (Table 1). However, review of scoring across stakeholder groups between Round 1 and 2 demonstrated two potential issues; i) that survey respondents were reluctant to score outcomes as ‘not important’ (score 1–3) resulting in an unmanageable number of ‘no consensus items’ that would need to be discussed at the consensus meeting and ii) an impractical number of ‘consensus in’ items for inclusion in a future COS. Following discussion with the steering group, it was considered unlikely that a further survey round would result in any further prioritisation. It was therefore decided post hoc to revise the definition of ‘consensus out’ (Table 1). The definition of ‘consensus in’ was retained, but thematically similar items were merged to create a smaller number of broader ‘summary’ outcome domains for inclusion in the final COS. Both the original and proposed summary outcome domains were reviewed and revised by the steering group prior to the consensus meeting, where the outcomes were presented for review and ratification.

All data were analysed in STATA V18 (www.stata.com).

Participant attrition between rounds was monitored. Weekly automatic reminder e-mails were sent to Round 1 participants who had either not completed or only partially completed the Round 2 survey to optimise participation.

### Phase III: Consensus meeting

The in-person consensus meeting was held in Barcelona, Spain on 14^th^ September 2024. Professional participants were purposively selected from those individuals who had completed both survey rounds and expressed an interest in attending the meeting. Sampling was based on stakeholder group, gender and geographical location to ensure a broad representation of views. An international group of experienced patient advocates were also invited to attend to ensure the discussions were patient focused. The meeting was facilitated by an independent chair (KC) who encouraged all participants to openly express their views. All participants were sent a meeting pack prior to the event which included a summary of the process and details of the consensus meeting format together with lists of the i) summary ‘consensus in’ and ii) ‘consensus out’ items for review, and iii) ‘no consensus’ items for discussion and voting at the meeting.

At the consensus meeting, a summary of the Delphi survey results was presented together with the patient and professional perspectives on the importance of a COS for context. ‘Consensus in’ and ‘consensus out’ items were then presented and participants given the opportunity for brief discussion to consider any objections to these items being included or excluded. Participants were then asked to vote to ratify the inclusion of outcome domains classified as ‘consensus in’ and exclusion of items classified as ‘consensus out’. Next, the ‘no consensus’ outcome domains were discussed in smaller multidisciplinary breakout groups. Each group was asked to identify up to three ‘no consensus’ items for inclusion in the COS and to provide a supporting justification. Each breakout group reported back verbally to the main group and all participants then voted on whether each ‘no consensus’ outcome domain was ‘very important’, ‘equivocal’ or ‘not important’ to include in the final COS. Domains voted as ‘very important’ by ≥70% of participants were included, either as separate outcomes or, if considered more appropriate, combined with existing ‘consensus in’ domains. All other items were discarded. Voting was conducted anonymously using MentiMeter software (www.mentimeter.com). The consensus meeting concluded with review and ratification of the final COS by meeting participants.

### Sample size

There is no standard sample size for consensus processes^18^ so the aim was to ensure good representation from key stakeholder groups across all geographical regions. A target sample size of 250–300 professionals for the survey and 20–25 participants for the consensus meeting was therefore agreed upon based on similar research^31,32^.

### Ethics

Ethical approval was obtained from the Queen’s University Belfast Faculty of Medicine, Health and Life Sciences Ethics committee (reference MHLS 23_167). Formal written consent was not obtained, as participation and attendance were taken as implied consent.

## Supplementary information

PRECEDENT_merged_supplementary materials

## Data Availability

All the data generated by this work has been included in the manuscript, tables and supplementary materials. No additional data are available.
