# Supplementary material for: A core outcome set for locoregional treatment reporting in neoadjuvant systemic breast cancer treatment trials
Source: NPJ Breast Cancer. 2025 Oct 28;11:116. doi: 10.1038/s41523-025-00824-w (PMC12568921; doi:10.1038/s41523-025-00824-w)
Supplement: Supplementary file 1 — PRECEDENT_merged_supplementary materials [file 41523_2025_824_MOESM1_ESM.pdf]

Supplementary table 1: Summary scores after Round 2 by participant group

SURGERY SECTION 1: TYPE OF SURGERY PLANNED BEFORE COMMENCING NST		Surgeons N=152		Radiation oncologists N=76		Other professionals N=103		Outcome following Round 2*
		Score 1-3	Score 7-9	Score 1-3	Score 7-9	Score 1-3	Score 7-9	
1	The number/proportion of patients considered eligible/ineligible for breast conserving surgery (BCS) before commencing NST and how BCS eligibility was assessed (e.g. surgeon assessment +/- imaging including type of imaging used)	11 (7.2)	123 (80.9)	17 (22.4)	47 (61.8)	7 (6.8)	80 (77.7)	In[b]
2	The number/proportion of patients planned for axillary node clearance/dissection (ANC/ALND) prior to commencing NST and how axillary staging was performed (e.g. clinical examination +/- imaging +/- biopsy)	7 (4.6)	124 (82.1)	17 (20.1)	49 (63.6)	5 (4.9)	84 (81.6)	In[b]
3	The number/proportion of patients having surgery before planned completion of NST (e.g., due to NST toxicity)	23 (15.1)	72 (47.4)	21 (27.6)	23 (30.3)	17 (16.5)	54 (52.4)	Out
4	The reasons for performing surgery before planned completion of NST (e.g. toxicity)	33 (21.7)	59 (38.8)	24 (31.6)	20 (26.3)	19 (18.6)	50 (49.0)	Out
5	A description of the management of patients having surgery before planned completion of NST (including type of surgery performed, if appropriate)	41 (27.0)	35 (23.0)	25 (33.3)	12 (16.0)	24 (23.5)	29 (28.4)	Out
6	The number/proportion of patients with progressive disease on NST	7 (4.6)	122 (80.3)	4 (5.2)	63 (81.8)	7 (6.8)	91 (88.4)	In[b]
7	The number/proportion of patients with progressive disease who do not have surgery and why	32 (20.9)	83 (54.3)	18 (23.4)	41 (53.3)	11 (10.6)	73 (70.2)	In[b]
**SURGERY SECTION 2: FACTORS INFLUENCING SURGICAL DECISION-MAKING FOLLOWING COMPLETION OF NST**								
8	The number/proportion of patients with germline mutations (e.g. BRCA1/2, PALB2)	18 (11.8)	92 (60.1)	17 (22.1)	28 (36.4)	9 (8.6)	69 (65.7)	No consensus
9	The number/proportion of patients with multifocal/multicentric disease and how multifocality/multicentricity was assessed (including the use of contrast enhanced imaging (with MRI or contrast enhanced mammography)	18 (11.8)	92 (60.5)	18 (23.4)	31 (40.3)	13 (12.5)	59 (56.7)	No consensus
10	How response to NST in the breast and axilla was assessed (e.g. clinical examination +/- imaging +/- biopsy and type of imaging/biopsies performed)	16 (10.5)	111 (73.0)	11 (14.3)	51 (66.2)	4 (3.9)	85 (82.5)	In[b]
11	How BCS eligibility was assessed following completion of NST	17 (11.3)	88 (58.7)	19 (25.0)	32 (42.1)	13 (12.4)	56 (53.3)	Out
12	How patient suitability for skin-sparing/nipple sparing mastectomy was assessed	37 (24.2)	49 (32.0)	25 (32.9)	12 (15.8)	20 (19.1)	23 (21.9)	Out
13	Reasons why patients are ineligible for BCS (after completion of NST)	19 (12.4)	97 (63.4)	18 (23.4)	29 (37.7)	14 (13.3)	60 (57.1)	No consensus
**SURGERY SECTION 3: TIMING OF SURGERY AFTER NST**								
14	The time from completion of NST to surgery	25 (16.5)	85 (55.9)	15 (19.5)	40 (52.0)	11 (10.7)	72 (69.9)	Out[a]
15	The time from start of NST to surgery	62 (41.1)	21 (13.9)	26 (34.2)	13 (17.1)	25 (24.3)	26 (25.2)	Out
16	The number/proportion of patients in whom surgery was delayed due to complications of NST	22 (14.5)	74 (48.7)	17 (22.1)	27 (35.1)	15 (14.9)	54 (53.5)	Out
17	The reasons for delays to surgery following completion of NST	31 (20.5)	49 (32.5)	22 (28.6)	18 (23.4)	19 (18.6)	38 (37.3)	Out
18	Whether there were any changes to the planned surgery (e.g. implant-based breast reconstruction not performed) due to complications of NST	37 (24.2)	54 (35.3)	24 (31.2)	13 (16.9)	22 (21.4)	25 (24.3)	Out
**SURGERY SECTION 4: BREAST SURGERY PERFORMED FOLLOWING NST**								
19	The type of breast surgery performed (mastectomy or BCS)	0 (0.0)	151 (99.3)	1 (1.3)	73 (96.1)	0 (0.0)	101 (99.0)	In[b]
20	The number/proportion of patients having oncoplastic breast conserving surgery and type performed (volume displacement/replacement)	18 (11.9)	87 (57.6)	18 (24.0)	38 (50.7)	13 (12.9)	51 (50.5)	Out
21	The number/proportion of patients undergoing mastectomy post NST who received immediate breast reconstruction and type of surgery performed	18 (11.9)	97 (64.2)	15 (19.7)	42 (55.3)	16 (15.7)	48 (47.1)	No consensus
22	The number/proportion of patients declining surgery after completion of NST	36 (23.8)	70 (46.4)	19 (25.0)	28 (36.8)	21 (20.6)	40 (39.2)	Out

		Surgeons N=152		Radiation oncologists N=76		Other professionals N=103		Outcome following Round 2*
		Score 1-3	Score 7-9	Score 1-3	Score 7-9	Score 1-3	Score 7-9	
23	The number/proportion of patients with a complete response to NST who avoided surgery including how response was assessed (radiologically and/or pathologically including details of how this was done e.g. multiple tumour bed biopsies)	17 (11.2)	114 (75.0)	5 (6.6)	62 (81.6)	9 (8.7)	74 (71.8)	In[b]
24	The weight of the surgical excision specimen	93 (61.2)	14 (9.2)	62 (82.7)	2 (2.7)	10 (9.7)	68 (66.0)	Out
25	Whether the pre-NST tumour footprint or the residuum (disease remaining after NST) was excised (i.e. was response-adjusted breast surgery performed?)	39 (26.0)	74 (49.3)	23 (30.7)	23 (30.7)	17 (16.5)	50 (48.5)	Out
26	The number/proportion of patients having bilateral surgery, including indication (malignancy vs risk reduction) and type of surgery performed	36 (23.7)	64 (42.1)	23 (30.7)	18 (24.0)	12 (11.7)	51 (49.5)	Out
**SURGERY SECTION 5: MARGIN ASSESSMENT AND MANAGEMENT OF INVOLVED/CLOSE MARGINS**								
27	The criteria used for defining clear margins (e.g. no tumour on ink)	10 (6.7)	124 (82.7)	8 (10.5)	60 (79.0)	5 (4.9)	89 (86.4)	In[b]
28	The number/proportion of patients with involved/close margins following attempted BCS post NST	7 (4.6)	123 (80.9)	7 (9.2)	62 (81.6)	6 (5.8)	83 (80.6)	In[b]
29	The criteria used for defining the rate of margin involvement/close margins (e.g. % with residual disease with involved/close margins vs % of all patients having BCS)	34 (22.5)	72 (47.7)	17 (23.4)	37 (48.7)	13 (13.0)	52 (52.0)	Out
30	The use of intraoperative margin assessment	51 (33.8)	28 (18.5)	32 (42.7)	9 (12.0)	25 (24.3)	22 (21.4)	Out
31	The method of intraoperative margin assessment used (e.g. specimen XR or other technology)	60 (39.5)	25 (16.5)	33 (43.4)	4 (5.3)	29 (28.4)	16 (15.7)	Out
32	The management of patients with involved/close margins (e.g. number/proportion of patients having re-excision of margins vs completion mastectomy)	10 (6.6)	119 (78.8)	8 (10.5)	52 (68.4)	11 (10.7)	66 (64.1)	In[b]
33	The number/proportion of patients choosing mastectomy due to involved/close margins after attempted BCS post NST	33 (22.0)	64 (42.7)	18 (23.7)	33 (42.4)	15 (15.6)	45 (43.7)	Out
34	The number/proportion of patients with involved/close margins after mastectomy and margin involved	34 (22.8)	65 (43.6)	13 (17.1)	44 (57.9)	14 (13.9)	46 (45.5)	Out
35	The number/proportion of patients with involved/close margins after completion of surgery	21 (13.8)	93 (61.2)	10 (13.2)	58 (76.3)	7 (6.8)	71 (68.9)	In[b]
36	The extent of margin involvement (e.g. number of margins involved and/or volume of disease present at the margin)	51 (34.0)	36 (24.0)	21 (27.6)	18 (23.7)	23 (22.3)	29 (28.2)	Out
37	The number/proportion of patients with involved/close margins who elected not to have further surgery and reasons why	47 (31.3)	34 (22.7)	37 (35.5)	17 (22.4)	25 (25.5)	29 (29.6)	Out
38	The number of procedures performed to achieve clear margins	45 (29.6)	57 (37.5)	28 (36.8)	13 (17.1)	26 (25.2)	26 (25.2)	Out
**SURGERY SECTION 6: AXILLARY SURGERY PERFORMED FOLLOWING NST**								
39	The type of axillary surgery performed (sentinel node biopsy/targeted axillary dissection/axillary sample/ANC/ALND)	0 (0)	148 (98.7)	0 (0)	75 (100.0)	1 (1.0)	101 (98.1)	In[b]
40	Details of how SLNB/TAD was performed (e.g. use of dual tracer, localisation of clipped node)	24 (15.9)	109 (72.2)	17 (22.7)	38 (50.7)	13 (12.8)	55 (53.9)	In[b]
41	The timing of axillary surgery (before start of NST or after completion of treatment)	31 (20.5)	77 (51.0)	7 (9.2)	47 (61.8)	10 (9.1)	69 (67.0)	No consensus
42	The total number of lymph nodes removed	20 (13.3)	98 (65.3)	6 (8.0)	57 (76.0)	8 (7.8)	83 (81.4)	In[b]
43	The number of involved lymph nodes removed and extent of involvement (isolated tumour cells (ITCs), micro or macrometastases)	7 (4.7)	137 (91.3)	1 (1.3)	73 (96.1)	2 (1.9)	97 (94.2)	In[b]
44	The number of negative lymph nodes removed	37 (24.5)	56 (37.1)	15 (20.0)	43 (57.3)	13 (12.8)	61 (59.8)	Out
45	The number of lymph nodes with evidence of treatment response (e.g. fibrosis)	27 (18.0)	90 (60.0)	11 (14.5)	46 (60.5)	13 (12.8)	67 (65.7)	No consensus
46	The size of sentinel lymph node(s)	112 (74.2)	10 (6.6)	40 (52.6)	8 (10.5)	53 (52.0)	14 (13.7)	Out
47	The nodal ratio (number of involved nodes/number of nodes removed)	44 (29.3)	43 (28.7)	21 (27.6)	22 (29.0)	22 (21.6)	40 (39.2)	Out

		Surgeons N=152		Radiation oncologists N=76		Other professionals N=103		Outcome following Round 2*
		Score 1-3	Score 7-9	Score 1-3	Score 7-9	Score 1-3	Score 7-9	
48	The number/proportion of patients undergoing completion ANC/ALND following a positive SLNB/TAD	12 (8.0)	123 (82.0)	5 (6.7)	63 (84.0)	5 (4.9)	90 (87.4)	In[b]
49	The number/proportion of patients NOT having completion ANC/ALND for involved nodes, extent of nodal involvement and receipt of alternative locoregional treatment (e.g. nodal radiotherapy)	14 (9.4)	112 (75.2)	7 (9.2)	61 (80.3)	8 (7.8)	81 (78.6)	In[b]
50	Rationale for type of axillary surgery performed (e.g. use of protocol/guidelines including whether response adjusted surgery performed)	42 (27.8)	54 (35.8)	23 (30.3)	24 (31.6)	20 (19.4)	42 (40.8)	Out
**SURGERY SECTION 7: SURGICAL COMPLICATIONS**								
51	The number/proportion of patients experiencing surgical complications (i.e. complications related to SURGERY such as wound infection) within 30 days of the procedure	25 (16.7)	82 (54.7)	16 (21.3)	27 (36.0)	17 (16.7)	51 (50.0)	Out
52	Details of the type/severity of surgical complications experienced	40 (26.9)	44 (29.5)	22 (29.3)	10 (13.3)	18 (17.7)	29 (28.4)	Out
53	The number/proportion of patients requiring readmission for complications of SURGERY within 30 days of the procedure	39 (26.0)	32 (21.3)	25 (32.9)	8 (10.5)	22 (21.6)	24 (23.5)	Out
54	The number/proportion of patients requiring reoperation for complications within 30 days of surgery	40 (26.7)	35 (23.3)	23 (30.7)	8 (10.7)	24 (23.5)	22 (21.6)	Out
55	The number/proportion of patients having breast reconstruction experiencing reconstruction related complications (e.g. implant or flap loss)	29 (19.5)	65 (43.6)	17 (23.0)	26 (35.1)	21 (20.6)	43 (42.2)	Out
56	The number/proportion of patients requiring treatment for surgical site infection within 30 days and intervention required (oral antibiotics/intravenous antibiotics/re-operation, washout +/- debridement	43 (29.1)	28 (18.9)	27 (35.5)	8 (10.5)	25 (24.8)	21 (20.8)	Out
57	The number/proportion of patients experiencing MEDICAL complications within 30 days of surgery (e.g. deep vein thrombosis, chest infection, myocardial infarction)	44 (29.5)	28 (18.8)	27 (35.5)	7 (9.2)	24 (23.5)	23 (22.6)	Out
**SURGERY SECTION 8: DE-ESCALATION OUTCOMES**								
58	The number/proportion of patients converting from BCS ineligible (requiring mastectomy) to BCS eligible following completion of NST	3 (2.0)	139 (92.7)	7 (9.2)	60 (79.0)	0 (0)	97 (96.0)	In[b]
59	The number/proportion of patients considered BCS eligible post NST having BCS	19 (12.7)	99 (66.0)	14 (18.4)	48 (63.2)	9 (9.0)	67 (67.0)	No consensus
60	The number/proportion of patients considered BCS eligible post NST having mastectomy and reasons why	24 (16.1)	85 (57.1)	16 (21.1)	40 (52.6)	11 (10.9)	65 (64.4)	Out[a]
61	The increase in the rate of BCS following NST	11 (7.3)	106 (70.7)	13 (17.1)	48 (63.2)	10 (9.9)	71 (70.3)	In[b]
62	The number/proportion of patients converting from cN+ to ycN0 post NST having response adjusted surgery (SNB/TAD)(i.e.,avoiding axillary clearance)	2 (1.3)	142 (95.3)	3 (4.0)	69 (90.8)	1 (1.0)	94 (92.2)	In[b]
63	The number/proportion of patients converting from cN+ to cN0 post NST having axillary clearance/dissection	11 (7.4)	114 (79.5)	11 (14.5)	52 (68.4)	6 (5.9)	83 (82.2)	In[b]
64	The number/proportion of patients in whom breast and/or axillary surgery was downstaged post NST	10 (6.7)	127 (84.7)	8 (10.5)	60 (79.0)	6 (5.9)	87 (86.1)	In[b]
65	The number/proportion of patients avoiding axillary surgery post NST	16 (10.7)	106 (71.1)	8 (10.5)	60 (79.0)	7 (6.9)	85 (83.3)	In[b]
**SURGERY SECTION 9: MISCELLANEOUS SURGICAL OUTCOMES**								
66	Length of hospital stay following surgery	93 (62.4)	9 (6.0)	50 (65.8)	2 (2.6)	41 (41.0)	11 (11.0)	Out
67	The cosmetic outcomes of surgery	38 (25.7)	38 (25.7)	23 (30.3)	13 (17.1)	24 (23.8)	24 (23.8)	Out
68	The patient-reported outcomes of surgery	24 (16.3)	80 (54.4)	19 (25.0)	31 (40.8)	13 (12.9)	50 (49.5)	Out
69	The involvement of multi-disciplinary team in treatment decision-making	36 (24.3)	69 (46.6)	21 (27.6)	37 (48.7)	17 (16.7)	60 (58.8)	Out

RADIATION THERAPY SECTION 1		Surgeons N=152		Radiation oncologists N=76		Other professionals N=103		Outcome following Round 2*
		Score 1-3	Score 7-9	Score 1-3	Score 7-9	Score 1-3	Score 7-9	
70	The number/proportion of patients receiving radiation therapy	2 (1.4)	138 (93.2)	3 (4.0)	71 (93.4)	1 (1.0)	98 (98.0)	In[b]
71	The indications for radiation therapy (i.e. TNM stage, ypTNM stage, other clinical or pathologic criteria)	14 (9.5)	111 (75.0)	5 (6.7)	66 (88.0)	6 (5.9)	82 (81.2)	In[b]
72	Whether radiation therapy protocols were used (including when there was no protocol)	31 (21.0)	69 (46.6)	14 (18.4)	44 (57.9)	14 (13.7)	52 (51.0)	Out
73	Description of local radiation therapy guidelines/policy for treatment following NST	39 (26.7)	60 (41.1)	11 (14.5)	43 (56.6)	15 (14.9)	51 (50.5)	Out
74	Change of practice/guidelines (radiation therapy indications) during study/trial	35 (23.7)	63 (42.6)	19 (25.0)	42 (55.3)	18 (17.7)	52 (51.0)	Out
75	The timing of the start of radiation therapy in relation to reconstructive surgery (number of days from surgery to radiation therapy)	29 (19.6)	68 (46.0)	21 (27.6)	41 (54.0)	13 (12.9)	62 (61.4)	Out[a]
76	The timing of the start of radiation therapy in relation to oncologic surgery (number of days from surgery to radiation therapy)	31 (21.1)	63 (42.9)	19 (25.3)	44 (58.7)	11 (10.8)	65 (63.7)	Out[a]
77	Reasons for delays to radiation therapy	34 (23.0)	47 (31.8)	21 (27.6)	27 (35.5)	19 (18.8)	38 (37.6)	Out
78	The number/proportion of patients declining/not receiving radiation therapy and reasons for this (patient choice, surgical complications etc)	36 (24.3)	52 (35.1)	20 (26.3)	40 (52.6)	16 (15.7)	45 (44.1)	Out
79	The sequencing of radiation therapy with surgery (before or after)	38 (25.7)	65 (43.9)	13 (17.1)	53 (69.7)	15 (14.7)	51 (50.0)	No consensus
80	Whether radiation therapy course was completed including reasons why and total dose received if course not completed	36 (24.5)	50 (34.0)	14 (18.4)	45 (59.2)	15 (14.9)	65 (64.4)	Out
81	Whether radiation therapy was given at the same time (within 1 week of) as adjuvant (post surgical) systemic therapy including details of systemic therapy	44 (29.9)	37 (25.2)	14 (18.4)	43 (56.6)	17 (16.7)	49 (48.0)	Out
82	Details of radiation therapy toxicity including grade	39 (26.4)	50 (33.8)	8 (10.5)	55 (72.4)	13 (12.9)	64 (63.4)	In[b]
**RADIATION THERAPY SECTION 2: VOLUME(S) IRRADIATED**								
83	Details of breast radiation target: partial breast, whole breast, chest wall	7 (4.8)	109 (74.7)	1 (1.4)	71 (96.0)	3 (3.0)	92 (91.1)	In[b]
84	Details of dose and fractionation to breast/chest wall targets	27 (18.4)	73 (49.7)	5 (6.6)	67 (88.2)	5 (4.9)	79 (74.5)	In[b]
85	Details of nodal radiation target(s) (L4 (supraclavicular fossa (SCF)), L3, L2, L1, internal mammary chain (IMC))	17 (11.6)	104 (71.2)	1 (1.3)	72 (94.7)	6 (5.9)	74 (73.3)	In[b]
86	Details of dose and fractionation to nodal targets	33 (22.5)	65 (44.2)	7 (9.2)	64 (84.2)	12 (11.8)	63 (61.8)	In[b]
87	Receipt of boost and indications	25 (16.9)	85 (57.4)	7 (9.2)	59 (77.6)	12 (11.8)	65 (63.7)	In[b]
88	Details of boost including targets, total dose and number of fractions and timing of boost: sequential or simultaneous integrated boost (SIB)	47 (31.8)	41 (27.7)	14 (18.4)	52 (68.4)	16 (15.8)	46 (45.5)	No consensus
89	Overall duration of radiotherapy	39 (26.9)	40 (27.6)	19 (25.0)	36 (47.4)	17 (16.7)	51 (50.0)	Out
90	Doses to 'organs at risk' (OARs) (heart/lung/contralateral breast)	51 (34.5)	29 (19.6)	13 (17.1)	42 (55.3)	23 (22.6)	34 (33.3)	Out
91	Coverage of target volumes (percentage of targets receiving prescription dose)	56 (38.1)	25 (17.0)	18 (24.3)	31 (41.9)	24 (23.8)	33 (32.7)	Out
92	Homogeneity of dose to target volumes (percentage "hotspot", volumetric hotspots)	62 (42.2)	14 (9.5)	23 (30.7)	11 (22.7)	28 (28.3)	21 (21.2)	Out
93	Modality of radiation therapy used (MV photons, KV photons, electrons, protons)	44 (30.1)	33 (22.6)	17 (22.4)	34 (44.7)	23 (22.8)	36 (35.6)	Out
94	Method used for radiotherapy planning	60 (41.1)	14 (9.6)	24 (32.0)	16 (21.3)	30 (29.7)	25 (24.8)	Out
95	Radiotherapy technique used (3D conformal, IMRT/VMAT)	56 (36.4)	30 (20.6)	20 (26.3)	37 (48.7)	20 (19.6)	45 (44.1)	Out
96	Beam energy (if applicable)	74 (50.3)	8 (5.4)	37 (48.7)	8 (10.5)	32 (31.4)	19 (18.6)	Out
97	Use of bolus and details including dose and schedule	61 (41.5)	12 (8.2)	24 (31.6)	24 (31.6)	31 (30.4)	23 (22.6)	Out
**RADIATION THERAPY SECTION 3: ON TREATMENT VERIFICATION**								
98	Type of images used for on treatment verification (MV, kV, cone beam CT scan (CBCT)	78 (53.1)	5 (3.4)	33 (43.4)	8 (10.5)	37 (36.6)	15 (14.9)	Out
99	Frequency of verification	87 (58.8)	4 (2.7)	36 (48.0)	8 (10.7)	42 (41.2)	11 (10.8)	Out
**RADIATION THERAPY SECTION 4: RADIATION THERAPY QUALITY ASSURANCE (QA)**								
100	Use of radiotherapy quality assurance as part of the trial/study processes	46 (31.3)	32 (21.8)	17 (23.4)	51 (67.1)	22 (21.6)	47 (46.1)	No consensus
101	Description of how radiotherapy quality assurance was done within the trial	57 (38.8)	30 (20.4)	19 (25.0)	41 (54.0)	27 (26.5)	36 (35.3)	Out

Carried forward for ratification at consensus meeting
[a]For ‘non consensus items’ only those important to between 60 and 70% of locoregional therapists were carried forward for discussion at the consensus meeting.
[b]31 ‘consensus in’ outcomes merged into 15 summary domains for inclusion in the final COS (see supplementary table 2)

Supplementary Table 2: ‘Consensus in’ outcomes after Delphi survey and summary ‘consensus in’ outcomes for inclusion in the final core outcome set

‘Consensus in’ surgical outcomes after Round 2 (n=23)		Summary surgical ‘consensus in’ items for inclusion in the final COS (n=9)
1	The number/proportion of patients considered eligible/ineligible for breast conserving surgery (BCS) before commencing NST and how BCS eligibility was assessed (e.g. surgeon assessment +/- imaging including type of imaging used)	*The type of breast and axillary surgery planned before starting NST*
2	The number/proportion of patients planned for axillary node clearance/dissection (ANC/ALND) prior to commencing NST and how axillary staging was performed (e.g. clinical examination +/- imaging +/- biopsy)	
6	The number/proportion of patients with progressive disease on NST	*Proportion of patients with disease which progressed during NST, including the proportion who did not have surgery and the reasons why*
7	The number/proportion of patients with progressive disease who do not have surgery and why	
10	How response to NST in the breast and axilla was assessed (e.g. clinical examination +/- imaging +/- biopsy and type of imaging/biopsies performed)	*How was response to NST in the breast and axilla assessed*
19	The type of breast surgery performed (mastectomy or BCS)	*Type of breast and axillary surgery performed after NST*
39	The type of axillary surgery performed (sentinel node biopsy/targeted axillary dissection/axillary sample/ANC/ALND)	
40	Details of how SLNB/TAD was performed (e.g. use of dual tracer, localisation of clipped node)	
27	The criteria used for defining clear margins (e.g. no tumour on ink)	*Definition of clear margins documented at trial level (recorded in protocol) and reported; proportion of patients with involved margins after initial and final surgery, and number of procedures required*
28	The number/proportion of patients with involved/close margins following attempted BCS post NST	
32	The management of patients with involved/close margins (e.g. number/proportion of patients having re-excision of margins vs completion mastectomy)	
35	The number/proportion of patients with involved/close margins after completion of surgery	
42	The total number of lymph nodes removed	*Total number of excised and involved lymph nodes, with extent of involvement*
43	The number of involved lymph nodes removed and extent of involvement (isolated tumour cells (ITCs), micro or macrometastases)	
48	The number/proportion of patients undergoing completion ANC/ALND following a positive SLNB/TAD	*Further axillary treatment in patients with ypN+ after SLNB/TAD*
49	The number/proportion of patients NOT having completion ANC/ALND for involved nodes, extent of nodal involvement and receipt of alternative locoregional treatment (e.g. nodal radiotherapy)	
58	The number/proportion of patients converting from BCS ineligible (requiring mastectomy) to BCS eligible following completion of NST	*Proportion of patients in whom breast and/or axillary surgery was downstaged*
61	The increase in the rate of BCS following NST	
62	The number/proportion of patients converting from cN+ to ycN0 post NST having response adjusted surgery (SNB/TAD)(i.e.,avoiding axillary clearance)	
64	The number/proportion of patients in whom breast and/or axillary surgery was downstaged post NST	
63	The number/proportion of patients converting from cN+ to cN0 post NST having axillary clearance/dissection	
65	The number/proportion of patients avoiding axillary surgery post NST	*The number/proportion of patients not having surgery to the breast and/or axilla, and how response was assessed*
23	The number/proportion of patients with a complete response to NST who avoided surgery including how response was assessed (radiologically and/or pathologically including details of how this was done e.g. multiple tumour bed biopsies)	
**‘Consensus in’ radiation therapy outcomes following Round 2 (n=8)**		**Summary radiation therapy outcomes for inclusion in the final COS (n=6)**
70	The number/proportion of patients receiving radiation therapy	*The proportion of patients receiving radiation therapy*
71	The indications for radiation therapy (i.e. TNM stage, ypTNM stage, other clinical or pathologic criteria)	*Indications for radiation therapy documented at trial level (recorded in protocol) and reported*
82	Details of radiation therapy toxicity including grade	*Side-effects of radiotherapy defined according to CTCAE*
83	Details of breast radiation target: partial breast, whole breast, chest wall	*Details of breast and nodal radiation targets*
85	Details of nodal radiation target(s) (L4 (supraclavicular fossa (SCF)), L3, L2, L1, internal mammary chain (IMC))	
84	Details of dose and fractionation to breast/chest wall targets	*Details of dose and fractionation to breast/chest wall and nodal areas*
86	Details of dose and fractionation to nodal targets	
87	Receipt of boost and indications	*Receipt of boost; indications for boost (at trial level)*

PRECEDENT
COS
Sample CRF

Notes

General notes/information for the Sample CRF

- The **titles of the forms** (e.g. form 1 „Initial Breast Cancer Assessment“), as well as the **variable prompts** (e.g. „Bilateral breast cancer?“) should only be seen as **suggestions**

- In the Sample CRF, **no codes** for variables (e.g. „Type of breast surgery planned before the start of NST “) or single choice variable answer options (e.g. „Breast Conserving Surgery (BCS)”, “Mastectomy”, etc.) **are included**, since these are usually dependent on sponsor-internal rules (standard operating procedures etc.). However, where possible, the use of codes as described by the Clinical Data Interchange Standards Consortium (**CDISC**, https://www.cdisc.org/) **is recommended**.

- In case **units** are documented in the Sample CRF (e.g. [mm] for tumor size or [Gy] for radiation dose), these **may be subject to change** depending on the study protocol. However, it **should always be clear** from the CRF, which unit is expected (either by pre-printing the unit to be used or by implementation of an additional variable with pre-defined unit choices in case of more than one possibility).

- Some answer options or complete variables in the Sample CRF are not seen as necessary for covering the 15 items of the Core Outcome Set (COS), but make sense to be included in most NST breast cancer study settings and are marked with **blue text color**.

- Depending on the software used for generating the eCRF for a study (i.e. the CDMS), various options may or may not be available to structure the CRF, e.g. a variable may be programmed to only be documentable in case a previous variable was documented in a certain way. Since not all used software is equal in this regard, **in the Sample CRF**, we have used **color-coded backgrounds** and **„If …“ statements** in combination with **indentations** to clarify that some variables have to be documented only under certain circumstances.
    - Example: „Breast cancer surgery performed?“
        - Answer „No“ → variable „If No, Reason breast surgery not performed” to be documented
        - Answer “Yes” → variable “If Yes, Type of initial breast surgery” and following variables to be documented

- In most CDMS, variables can either be programmed as **individual variables** on a Form, **or** as part of a group of variables within a **table-format**. The table format may sometimes be less clear compared with individual variables, especially in case multiple variables are packed into a single table, but an advantage is that multiple lines of grouped variables (i.e. individual log-lines or rows) can be documented so that the CRF programmer does not need to know beforehand how many variables of a certain type to include on a Form.
    - Example: Breast cancer examinations: Multiple examinations (e.g. clinical, imaging) for multiple lateralities and/or lesions may be documented and therefore, the table-format was used in the Sample CRF instead of pre-printing 20 or more copies of all variables included in the table to make sure that all measured lesions can also be documented.

- In the **Sample CRF**, we have included the table format wherever we find it easier to document, especially in case an unknown number of loglines is expected. However, the presented **design** is just to be **seen as suggestion** and all variables in tables could theoretically be programmed as (multiple) individual variables and all individual variables could be included in tables.

- A general issue in breast cancer studies is the possibility of **bilateral** and/or multifocal/multicentric (primary) **tumors**. To make sure that bilateral tumors can be docmented adequately and that it is always clear which measurement belongs to which laterality, we have **included most variables** in the Sample CRF **twice** (or with the additional option to document the laterality in case of variables in table format).
    - **Note**: In case of the **surgery** form variables, it may make sense to allow documentation **for both lateralities** also in case of unilateral breast cancer since the number of precautionary bilateral mastectomies has increased over the last years.

- In case of **multicentric** and/or **multifocal** breast cancers, depending on the study design, often only one lesion (per laterality), e.g. the **largest lesion** or the most metabolically active lesion, may be of interest and termed „target lesion". For such cases, optional variables for target lesions are included in the Sample CRF.

- For many variables, **optional answers „Unknown"** and/or **„Not evaluable"** and/or **„Not assessed"** are included since other pre-defined answers might not be correct and documenting one of the mentioned options is unambiguous compared to the alternative of not documenting a variable at all.

- Furthermore, for several variables the **answer option „Other"** is included with an optional free text field for further specification.

- **Date variables** may not always be 100% necessary to fulfill the 15 COS items, but since most of the time the dates of performed procedures etc. should be known, we have included them where deemed reasonable.
    - Note: The date format used should always be unambiguous (e.g. DD-MMM-YYYY), but in case that is not possible (due to technical reasons), the **format to be used should be specified on the CRF** (in the Sample CRF we have included the format in brackets for each date variable)

- The **allocation of variables to certain Forms** is, in general, not defined by any standard, i.e. any variable from any of the Sample CRF Forms can be switched to any other Form (or, for that matter, a completely different Form) and Forms can be split up or merged in any way. However, to promote easy documentation and prevent confusion for data documentation personnel, **in the Sample CRF**, we aimed to logically order the Forms and the variables within the Forms in a clear and unambiguous way.

    - Note: **The Sample CRF only includes variables seen as relevant for the 15 PRECEDENT COS items**, but a complete CRF of course includes more than just these Forms/ variables (**for standard endpoints like PFS, OS, IDFS etc., Forms including death details and tumor recurrence/new lesions are of course also necessary in addition to any study-specific endpoint forms/variables**).

      The additional Forms/variables of a complete CRF may be arranged anywhere before, in-between or after the Forms presented in the Sample CRF (and more variables might be included, depending on the specific study protocol). Furthermore, the **suggested prompts and answer options** within the Sample CRF should always be understood as suggestions and **may have to be adapted according to** the specific **study protocol**.

COS Item representation on the sample CRF

1. "The type of breast and axillary surgery planned before starting NST"
   → Form 2 (Planned Surgery before NST)
2. "The proportion of patients who did not have surgery after NST due to disease progression, treatment toxicities or other comorbidities"
   → Form 4 (Surgical Data Collection after NST)
3. "The number/proportion of patients with a complete response to NST not having surgery to the breast and/or axilla, and how response was assessed"
   → Form 1 (Initial Breast Cancer Assessment) – Baseline
   → Form 3 (Disease Response after NST) – Clinical/Radiological Response
   → Form 4 (Surgical Data Collection after NST) – Surgery
   → Form 5 (Pathological Staging and Response) – Pathological Response
4. "How response to NST in the breast and axilla was assessed"
   → Form 3 (Disease Response after NST)
5. "Type of initial breast and axillary surgery performed after NST"
   → Form 4 (Surgical Data Collection after NST)
6. "Proportion of patients with involved margins after initial and final surgery, and number of procedures required"
   → Form 4 (Surgical Data Collection after NST) & Trial protocol (definition of clear margins to be stated in protocol)
7. "Total number of excised and involved axillary lymph nodes, with extent of involvement"
   → Form 4 (Surgical Data Collection after NST)
   → Form 5 (Pathological Staging and Response)
8. "Further axillary treatment in patients with ypN+ disease after SLNB/TAD"
   → Form 4 (Surgical Data Collection after NST)
9. "Proportion of patients in whom breast and/or axillary surgery was downstaged"
   Derived from data entered on:
   → Form 2 (Planned Surgery before NST) &
   → Form 4 (Surgical Data Collection after NST)
10. "The proportion of patients having radiation therapy"
    → Form 6 (Adjuvant Radiotherapy)
11. "Indications for radiation therapy at trial level"
    →Trial protocol
12. "Details of breast/chest wall and nodal targets"
    → Form 6 (Adjuvant Radiotherapy)
13. "Details of dose and fractionation to breast/chest wall and nodal areas"
    → Form 6 (Adjuvant Radiotherapy)
14. "Receipt of boost; indications for boost (at trial level)"
    → Form 6 (Adjuvant Radiotherapy) & Trial protocol (for indications)
15. "Morbidity of locoregional treatments (short and long term as defined in protocol)"
    → Form 7 (Adverse Events) and as defined in Trial protocol

Sample CRF

Form 1:

Initial Breast Cancer Assessment

**Bilateral breast cancer?**	- Yes<br>- No

Document a single row for each affected laterality (i.e. 2 rows in case of bilateral disease):

Laterality	Multifocal breast cancer?	Multicentric breast cancer?
- Left<br>- Right	- Yes<br>- No<br>- Unknown	- Yes<br>- No<br>- Unknown
…	…	…

Examinations:

List each performed examination as individual table row (examinations not documented are seen as not performed):

Laterality	Examination performed	If Other, specify	If Imaging, specify	If Other, specify	Date of examination *(DDMMMYYYY)*
- Left<br>- Right	- Clinical Breast Examination (CBE)/palpation<br>- Imaging<br>- Other	*[Free text field]*	- Mammography<br>- Contrast-Enhanced Spectral Mammography (CESM)<br>- Ultrasound<br>- MRI<br>- Other	*[Free text field]*	*[Date field]*
…	…	…	…	…	

Table continued:

Location	If Other, specify	Tumor size long axis [mm]	Tumor size short axis [mm]	Target lesion
- Breast – upper outer quadrant<br>- Breast – upper inner quadrant<br>- Breast – lower outer quadrant<br>- Breast – lower inner quadrant<br>- Breast – upper quadrants<br>- Breast – lower quadrants<br>- Breast – outer quadrants<br>- Breast – inner quadrants<br>- Internal mammary lymph node<br>- Infraclavicular lymph node<br>- Supraclavicular lymph node<br>- Axillary lymph node<br>- Other	*[Free text field]*	*[Number field]*	*[Number field]*	- Yes<br>- No
…	…	…	…	…

Form continued on next slide

Form 1 - continued:

Clinical TNM Staging:

Document a single row for each affected laterality (i.e. 2 rows in case of bilateral disease):

Laterality	cT stage	cN stage	M stage	Grade
- Left<br>- Right	- cTX<br>- cT0<br>- cTis<br>- cT1<br>- cT1mi<br>- cT1a<br>- cT1b<br>- cT1c<br>- cT2<br>- cT3<br>- cT4<br>- cT4a<br>- cT4b<br>- cT4c<br>- cT4d	- cNX<br>- cN0<br>- cN1<br>- cN1mi<br>- cN2<br>- cN2a<br>- cN2b<br>- cN3<br>- cN3a<br>- cN3b<br>- cN3c	- M0<br>- M0(i+)<br>- M1	- GX<br>- G1<br>- G2<br>- G3
...	...	...	...	...

Form 2:

Planned Surgery before NST

Document a single row for each affected laterality (i.e. 2 rows in case of bilateral disease):

Laterality	Type of breast surgery planned before the start of NST	Type of axillary surgery planned before the start of NST
- Left<br>- Right	- Breast-Conserving Surgery (BCS)<br>- Mastectomy<br>- Not evaluable<br>- Unknown	- Sentinel Lymph Node Biopsy (SLNB)<br>- Targeted Axillary Dissection (TAD)<br>- Axillary Lymph Node Dissection (ALND)<br>- Not evaluable<br>- Unknown
…	…	…

Form 3:

How was disease response after NST assessed

Tumor assessments:

List each performed examination as individual table row (examinations not documented are seen as not performed):

Laterality	Examination performed	If Other, specify	If Imaging, specify	If Other, specify	Date of examination *(DDMMMYYYY)*
- Left<br>- Right	- Clinical Breast Examination (CBE)/palpation<br>- Imaging<br>- Other	*[Free text field]*	- Mammography<br>- Contrast-Enhanced Spectral Mammography (CESM)<br>- Ultrasound<br>- MRI<br>- CT Scan<br>- PET Scan<br>- PET-CT<br>- Other	*[Free text field]*	*[Date field]*
…	…	…	…	…	…

Table continued:

Location	If Other, specify	Tumor size long axis [mm]	Tumor size short axis [mm]	Target lesion?
- Breast – upper outer quadrant<br>- Breast – upper inner quadrant<br>- Breast – lower outer quadrant<br>- Breast – lower inner quadrant<br>- Breast – upper quadrants<br>- Breast – lower quadrants<br>- Breast – outer quadrants<br>- Breast – inner quadrants<br>- Internal mammary lymph node<br>- Infraclavicular lymph node<br>- Supraclavicular lymph node<br>- Axillary lymph node<br>- Other	*[Free text field]*	*[Number field]*	*[Number field]*	- Yes<br>- No
…	…	…	…	…

Form continued on next slide

Biopsy:

List each performed biopsy as individual table row (biopsies not documented are seen as not performed):

Laterality	Biopsy performed?	If Yes, specify	If Other, specify	Location
- Left<br>- Right	- Yes<br>- No<br>- Unknown	- Fine-needle aspiration (FNA)<br>- Core-needle biopsy<br>- Skin-punch biopsy<br>- Other	*[Free text field]*	- Breast – upper outer quadrant<br>- Breast – upper inner quadrant<br>- Breast – lower outer quadrant<br>- Breast – lower inner quadrant<br>- Breast – upper quadrants<br>- Breast – lower quadrants<br>- Breast – outer quadrants<br>- Breast – inner quadrants<br>- Internal mammary lymph node<br>- Infraclavicular lymph node<br>- Supraclavicular lymph node<br>- Axillary lymph node<br>- Other
…	…	…	…	…

Table continued:

If Other, specify	Date of examination *(DDMMMYYYY)*	Target lesion?	Residual disease?
[Free text field]	*[Date field]*	-) Yes<br>-) No	- Yes<br>- No<br>- Unknown
…	…	…	…

Overall Response:

Note: *Depending on the study protocol, additional response evaluations might be recorded (e.g. metabolical response); pathological response should be documented following surgical data collection, see form 5*

Document a table row for each response type (clinical, radiological) for breast and axilla for each applicable laterality:

Laterality	Response Location	Response Type	Overall Response
- Left<br>- Right	- Breast<br>- Axilla	- Clinical<br>- Radiological<br>- Metabolical	- Complete Response (CR)<br>- Partial Response (PR)<br>- Stable Disease (SD)<br>- Progressive Disease (PD)<br>- Not evaluable<br>- Not assessed<br>- Unknown
…	…	…	…

Form 4:

Surgical Data Collection after NST

Laterality:
- Left
- Right

Breast cancer surgery performed?
- Yes
- No
- Not applicable

If No,
Reason breast surgery not performed:
- Excellent response to NST
- Locally advanced disease/inoperable
- Progressive disease
- Treatment toxicities
- Other co-morbidities
- Unknown
- Other

If other reason,
Specify:
[Free text field]

If Yes,
Type of initial breast surgery:
- Breast-Conserving Surgery (BCS)
- Mastectomy
- Unknown

Date of initial breast surgery: *[Date field]* *[DDMMMYYYY]*

If BCS,
Surgical margin clear after initial breast surgery?
- Yes
- No
- Unknown

Additional surgery performed?
- Yes
- No
- Unknown

If Yes,
Type of final breast surgery performed:
- Breast-Conserving Surgery (BCS)
- Mastectomy
- Unknown

Surgical margin clear after final breast surgery performed?
- Yes
- No
- Unknown

Total number of additional breast surgeries performed *[Number field]*

Form continued on next slide

Form 4 - continued:

Axillary surgery performed?

- Yes
- No
- Not applicable

If No,
Reason axillary surgery not performed:

- No or low lymph node involvement
- Excellent response to NST
- Locally advanced disease/inoperable
- Progressive disease
- Metastatic disease
- Treatment toxicities
- Other co-morbidities
- Unknown
- Other

If other reason,
Specify: *[Free text field]*

If Yes,
Type of initial axillary surgery:

- Sentinel Lymph Node Biopsy (SLNB)
- Targeted Axillary Dissection (TAD)
- Axillary Lymph Node Dissection (ALND)
- Unknown
- Other

If other,
Specify: *[Free text field]*

Date of initial axillary surgery: *[Date field]* *[DDMMMYYYY]*

Number of positive axillary lymph nodes: *[Number field]*

Number of removed axillary lymph nodes: *[Number field]*

If SLNB or TAD,
Further treatment after SLNB/TAD:

- Axillary Lymph Node Dissection (ALND)
- Radiotherapy (RT)
- None
- Unknown

Form continued on next slide

Form 4 - continued:

Document for second laterality:

Laterality:
- Left
- Right

Breast cancer surgery performed?
- Yes
- No
- Not applicable

If No,
Reason breast surgery not performed:
- Excellent response to NST
- Locally advanced disease/inoperable
- Progressive disease
- Treatment toxicities
- Other co-morbidities
- Unknown
- Other

If other reason,
Specify: [Free text field]

If Yes,
Type of initial breast surgery:
- Breast-Conserving Surgery (BCS)
- Mastectomy
- Unknown

Date of initial breast surgery: [Date field] [DDMMMYYYY]

If BCS,
Surgical margin clear after initial breast surgery?
- Yes
- No
- Unknown

Additional surgery performed?
- Yes
- No
- Unknown

If Yes,
Type of final breast surgery performed:
- Breast-Conserving Surgery (BCS)
- Mastectomy
- Unknown

Surgical margin clear after final breast surgery performed?
- Yes
- No
- Unknown

Total number of additional breast surgeries performed [Number field]

Form continued on next slide

Form 4 - continued:

Axillary surgery performed?

- Yes
- No
- Not applicable

If No,
Reason axillary surgery not performed:

- No or low lymph node involvement
- Excellent response to NST
- Locally advanced disease/inoperable
- Progressive disease
- Metastatic disease
- Treatment toxicities
- Other co-morbidities
- Unknown
- Other

If other reason,
Specify: *[Free text field]*

If Yes,
Type of initial axillary surgery:

- Sentinel Lymph Node Biopsy (SLNB)
- Targeted Axillary Dissection (TAD)
- Axillary Lymph Node Dissection (ALND)
- Unknown
- Other

If other,
Specify: *[Free text field]*

Date of initial axillary surgery: *[Date field]* *[DDMMMYYYY]*

Number of positive axillary lymph nodes: *[Number field]*

Number of removed axillary lymph nodes: *[Number field]*

If SLNB or TAD,
Further treatment after SLNB/TAD:

- Axillary Lymph Node Dissection (ALND)
- Radiotherapy (RT)
- None
- Unknown

Form 5:

Pathological Staging and Response

Pathological TNM Staging:

Document a single row for each affected laterality (i.e. 2 rows in case of bilateral disease):

Laterality	p or yp stage?	T stage	N stage	M stage	Grade
- Left<br>- Right	- p (upfront surgery)<br>- yp (neoadjuvant treatment)	- TX<br>- T0<br>- Tis<br>- T1<br>- T1mi<br>- T1a<br>- T1b<br>- T1c<br>- T2<br>- T3<br>- T4<br>- T4a<br>- T4b<br>- T4c<br>- T4d	- NX<br>- N0<br>- N0(i+)<br>- N0(mol+)<br>- N1<br>- N1mi<br>- N1a<br>- N1b<br>- N1c<br>- N2<br>- N2a<br>- N2b<br>- N3<br>- N3a<br>- N3b<br>- N3c	- M0<br>- M0(i+)<br>- M1	- GX<br>- G1<br>- G2<br>- G3
…	…	…	…	…	…

Pathological Response:

Document a single row for each affected laterality (i.e. 2 rows in case of bilateral disease):

Laterality	Overall pathological response to NST	RCB score	RCB class
- Left<br>- Right	- Complete Response (CR)<br>- Partial Response (PR)<br>- No evidence of response<br>- Not evaluable<br>- Not assessed<br>- Unknown	*[Number field]*	- RCB 0<br>- RCB I<br>- RCB II<br>- RCB III<br>- Not evaluable<br>- Not assessed<br>- Unknown
…	…	…	…

Form 6:

Adjuvant Radiotherapy

Laterality:
- Left
- Right

Adjuvant radiotherapy performed?
- Yes
- No
- Unknown

If Yes,
Start date of radiotherapy: [Date field] *[DDMMMYYYY]*

End date of radiotherapy: [Date field] *[DDMMMYYYY]*

Radiation of breast or chest wall performed?
- Yes
- No
- Unknown

If Yes,
Type of breast/chest wall radiotherapy:
- Whole Breast Irradiation (WBI)
- Accelerated Partial Breast Irradiation (APBI)
- Intraoperative Radiotherapy (IORT)
- Chest Wall Irradiation
- Other
- Unknown

If other reason,
Specify: *[Free text field]*

Boost (tumor bed/chest wall) received?
- Yes
- No
- Unknown

If no,
Number of total fractions: *[Number field]*

Dose per fraction: *[Number field]* [Gy]

If yes,
Type of boost received:
- Simultaneous Integrated Boost (SIB)
- Sequential Boost (SEB)
- Unknown

If Simultaneous Integrated Boost (SIB),
Number of total fractions: *[Number field]*

Dose per fraction, tumor bed: *[Number field]* [Gy]

Dose per fraction, surrounding tissue: *[Number field]* [Gy]

If Sequential Boost (SEB),
Number of fractions, excluding boost: *[Number field]*

Dose per fraction, excluding boost: *[Number field]* [Gy]

Number of boost fractions: *[Number field]*

Dose per boost fraction: *[Number field]* [Gy]

Form continued on next slide

Form 6 - continued:

Radiation of lymph node regions performed?
- Yes
- No
- Unknown

If Yes,

Radiation of Axilla Level I
- Yes
- No
- Unknown

Radiation of Axilla Level II
- Yes
- No
- Unknown

Radiation of Axilla Level III
- Yes
- No
- Unknown

Radiation of medial supraclavicular Region (Level IV)
- Yes
- No
- Unknown

Radiation of parasternal/internal mammary region
- Yes
- No
- Unknown

Number of fractions: *[Number field]*

Dose per fraction: *[Number field]* [Gy]

Form continued on next slide

Form 6 - continued:

If bilateral breast cancer, document second laterality:

Laterality:
- Left
- Right

Adjuvant radiotherapy performed?
- Yes
- No
- Unknown

If Yes,
Start date of radiotherapy: *[Date field]* *[DDMMYYYY]*

End date of radiotherapy: *[Date field]* *[DDMMYYYY]*

Radiation of breast or chest wall performed?
- Yes
- No
- Unknown

If Yes,
Type of breast/chest wall radiotherapy:
- Whole Breast Irradiation (WBI)
- Accelerated Partial Breast Irradiation (APBI)
- Intraoperative Radiotherapy (IORT)
- Chest Wall Irradiation
- Other
- Unknown

If other reason,
Specify: *[Free text field]*

Boost (tumor bed/chest wall) received?
- Yes
- No
- Unknown

If no,
Number of total fractions: *[Number field]*

Dose per fraction: *[Number field]* [Gy]

If yes,
Type of boost received:
- Simultaneous Integrated Boost (SIB)
- Sequential Boost (SEB)
- Unknown

If Simultaneous Integrated Boost (SIB),
Number of total fractions: *[Number field]*

Dose per fraction, tumor bed: *[Number field]* [Gy]

Dose per fraction, surrounding tissue: *[Number field]* [Gy]

If Sequential Boost (SEB),
Number of fractions, excluding boost: *[Number field]*

Dose per fraction, excluding boost: *[Number field]* [Gy]

Number of boost fractions: *[Number field]*

Dose per boost fraction: *[Number field]* [Gy]

Form continued on next slide

Form 6 - continued:

Radiation of lymph node regions performed?
- Yes
- No
- Unknown

If Yes,

Radiation of Axilla Level I
- Yes
- No
- Unknown

Radiation of Axilla Level II
- Yes
- No
- Unknown

Radiation of Axilla Level III
- Yes
- No
- Unknown

Radiation of medial supraclavicular Region (Level IV)
- Yes
- No
- Unknown

Radiation of parasternal/internal mammary region
- Yes
- No
- Unknown

Number of fractions: *[Number field]*

Dose per fraction: *[Number field]* [Gy]

Form 7:

Adverse Events

***Note:** This form is intended as repeating form, i.e. a new form is to be documented for each unique Adverse Event/ morbidity as defined in the study protocol*

Were any Adverse Events experienced?	- Yes<br>- No<br>- Unknown
Adverse Event Term (Verbatim):	*[(Coded) Free text field]*

List each new occurrence or grade change of the same Adverse Event as individual row in the table row:

**Event Grade (NCI CTCAE grade)**	**Onset date** *(DDMMMYYYY)*	**Outcome of Adverse Event**	**Intermittent AE?**	**Resolution date** *(DDMMMYYYY)*
- Grade 1<br>- Grade 2<br>- Grade 3<br>- Grade 4<br>- Grade 5	[Date field]	- Recovered/Resolved<br>- Recovered/Resolved with sequelae<br>- Recovering/Resolving<br>- Not Recovered/Not Resolved<br>- Not recovered/Not resolved at end of reporting period<br>- Fatal<br>- Unknown	- Yes<br>- No	[Date field]
…	…	…	…	…

Table continued:

**Relationship to breast cancer/axilla surgery**	**Relationship to radiotherapy**	**Relationship to [medication/ therapy]**	**Action taken ([medication/therapy])**	**Relationship to [medical device (IVD)]**
- Yes<br>- No<br>- Not applicable	- Yes<br>- No<br>- Not applicable	- Yes<br>- No<br>- Not applicable	- None<br>- Modified<br>- Delayed<br>- Interrupted<br>- Modified and interrupted<br>- Modified and delayed<br>- Modified, delayed and interrupted<br>- Delayed and interrupted<br>- Drug Withdrawn	- Not related<br>- Possible<br>- Probable<br>- Causal relationship
…	…	…	…	…

Table continued:

**Adverse Event of Special Interest (AESI)?**	**Serious Adverse Event (SAE)?**	**Medication taken due to this AE?**	**Procedure/ surgery due to this AE?**	**If yes, Type of procedure/ surgery**	**Hospitalization (initial or prolonged) due to this AE?**	**Withdrawn from study due to this AE?**
- Yes<br>- No	- Yes<br>- No	- Yes<br>- No	- Yes<br>- No	[Free text field]	- Yes<br>- No	- Yes<br>- No
…	…	…	…	…	…	…
